# A novel ΔNp63-dependent immune mechanism improves prognosis of HPV-related head and neck cancer

**DOI:** 10.3389/fimmu.2023.1264093

**Published:** 2023-10-25

**Authors:** Jana Mourtada, Christelle Lony, Anaïs Nicol, Justine De Azevedo, Cyril Bour, Christine Macabre, Patrick Roncarati, Sonia Ledrappier, Philippe Schultz, Christian Borel, Mickaël Burgy, Bohdan Wasylyk, Georg Mellitzer, Michaël Herfs, Christian Gaiddon, Alain C. Jung

**Affiliations:** ^1^ Laboratoire de Biologie Tumorale, Institut de cancérologie Strasbourg Europe, Strasbourg, France; ^2^ Université de Strasbourg-Inserm, UMR_S 1113 IRFAC, Laboratory « Streinth », Strasbourg, France; ^3^ Laboratoire de Radiobiologie, Institut de cancérologie Strasbourg Europe, Strasbourg, France; ^4^ Tumorothèque du Centre Paul Strauss, Centre Paul Strauss, Strasbourg, France; ^5^ Laboratory of Experimental Pathology, GIGA-Cancer, University of Liège, Liège, Belgium; ^6^ Hôpitaux Universitaires de Strasbourg, Department of Otorhinolaryngology and Head and Neck Surgery, Strasbourg, France; ^7^ Department of Medical Oncology, Institut de cancérologie Strasbourg Europe, Strasbourg, France; ^8^ Department of Functional Genomics and Cancer, Institut de Génétique et de Biologie Moléculaire et Cellulaire (IGBMC), Illkirch-Graffenstaden, France; ^9^ Institut National de la Santé et de la Recherche Médicale (INSERM) U 1258, Illkirch-Graffenstaden, France; ^10^ Centre Nationale de la Recherche Scientifique (CNRS) UMR 7104, Illkirch-Graffenstaden, France; ^11^ Université de Strasbourg, Strasbourg, France

**Keywords:** ΔNp63, immune response, human papillomavirus, oropharyngeal squamous cell carcinoma, tumor microenvironment

## Abstract

**Background:**

Deconvoluting the heterogenous prognosis of Human Papillomavirus (HPV)-related oropharyngeal squamous cell carcinoma (OSCC) is crucial for enhancing patient care, given its rapidly increasing incidence in western countries and the adverse side effects of OSCC treatments.

**Methods:**

Transcriptomic data from HPV-positive OSCC samples were analyzed using unsupervised hierarchical clustering, and clinical relevance was evaluated using Kaplan-Meier analysis. HPV-positive OSCC cell line models were used in functional analyses and phenotypic assays to assess cell migration and invasion, response to cisplatin, and phagocytosis by macrophages *in vitro*.

**Results:**

We found, by transcriptomic analysis of HPV-positive OSCC samples, a ΔNp63 dependent molecular signature that is associated with patient prognosis. ΔNp63 was found to act as a tumor suppressor in HPV-positive OSCC at multiple levels. It inhibits cell migration and invasion, and favors response to chemotherapy. RNA-Seq analysis uncovered an unexpected regulation of genes, such as DKK3, which are involved in immune response-signalling pathways. In agreement with these observations, we found that ΔNp63 expression levels correlate with an enhanced anti-tumor immune environment in OSCC, and ΔNp63 promotes cancer cell phagocytosis by macrophages through a DKK3/NF-κB-dependent pathway.

**Conclusion:**

Our findings are the first comprehensive identification of molecular mechanisms involved in the heterogeneous prognosis of HPV-positive OSCC, paving the way for much-needed biomarkers and targeted treatment.

## Graphical Abstract

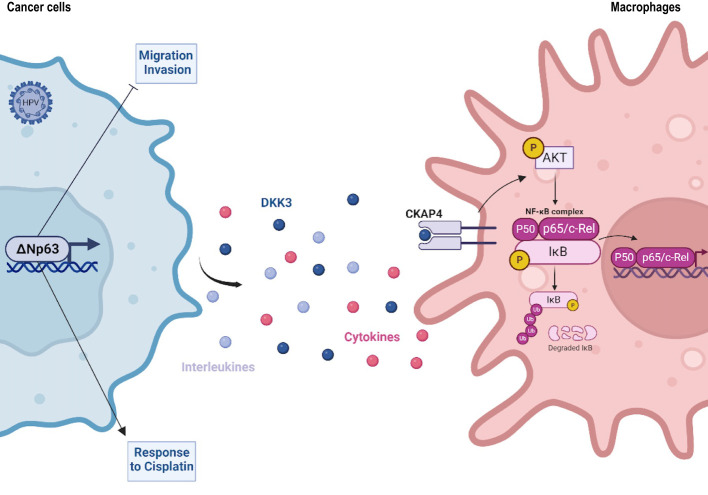

## 1 Introduction

There is a worldwide significant increase in the prevalence of Human Papillomavirus (HPV)-related oropharyngeal squamous cell carcinoma (OSCC) (1), which is now considered to be clinically distinct, with improved prognosis and specific biological features [see (2) and references therein]. Compared to HPV-negative Head and Neck Squamous Cell Carcinoma (HNSCC), the rate of metastatic spread is similar, but the target organs are different (3, 4). HPV tumor status is now included in the 8^th^ edition for TNM staging and risk stratification (5). However, HPV-related OSCC is still treated therapeutically as HPV-negative HNSCC, with concomitant platinum-based chemoradiation therapy that causes severe acute and late toxicities (6, 7). Quality of life could be improved with therapeutic de-escalation, but there are no prognostic biomarkers to identify suitable patients (8). There is growing evidence for clinical heterogeneity of HPV-positive OSCC. For example, the molecularly defined “mesenchymal/inflamed” subgroup has longer overall survival than the “classical” (xenobiotic metabolism) subgroup (9). Higher expression of CD8 and/or PD-L1 in HPV-related OSCC tumors has been associated with improved prognosis (10, 11). However, the molecular mechanisms that drive improved prognosis need to be discovered.

We now report novel molecular processes that underly OSCC heterogeneous prognosis. They involve a differential expression of the ΔNp63 transcription factor and its antitumor activities. ΔNp63 is a member of the p53 family of transcription factors (12), which plays a major role in various cancers and is targeted by novel therapeutic approaches (13, 14). ΔNp63 lacks the N-terminal transactivation domain of TAp63, and is abundantly expressed in oral epithelium (15), as well as HNSCC (16), where it plays an oncogenic role in chemically-driven skin carcinogenesis (17). Here we show that ΔNp63 has a key role in a subset of HPV-related OSCC and significantly affects various cellular processes (*e.g*., cell migration/invasion; response to cisplatin) that could account for differences in patient prognosis. Most importantly, our data provides evidence that ΔNp63 in cancer cells induces the expression of diffusible immunomodulators by cancer cells, including Dickkopf-related protein 3 (DKK3), that activates the NF-κB signalling pathway in immune cells.

## 2 Materials and methods

### 2.1 Patient characteristics and tumor samples

Eight HPV-positive patients with OSCC samples and available transcriptomic data were included in this study. Patient description is shown in **Supplementary Table S1**. Patients’ mean age is 59 years old and median follow-up is 130 months. Patients underwent initial surgery (Sainte Barbe clinic, Strasbourg) followed by chemoradiotherapy (Centre Paul Strauss) between 1993 and 1995. Transcriptomic data was previously generated using an Affymetrix GeneChip approach, as described in (18).

Gene expression analysis, immunohistochemistry and survival analyses were carried out on a cohort of 77 patients that were treated in Strasbourg, France (N=34; they include the 8 HPV-positive OSCC that generated the transcriptomic data), and Liège, Belgium (N=43) between 1993 and 2014. Patient description is shown in **Supplementary Table S1**. Patients’ mean age is 60 years old and median follow-up is 37 months.

In all cases, tumor samples were collected at the time of surgery with the patients’ informed consent, and stored in the “Centre Paul Strauss” tumor bank (Strasbourg, France) or the “Biothèque Hospitalo-Universitaire de Liège” (Liège, Belgium). A fragment was taken near the advancing edge of the primary tumor (avoiding its necrotic center), immediately frozen in liquid nitrogen, and stored at −70°C. The rest of the tumor was fixed in 6% buffered formalin and embedded in paraffin for histopathological analysis. Examination of sections adjacent to each tumor fragment showed that the percentage of tumor cells was over 70%. The TNM system of the UICC was used for tumor‐node‐metastasis staging (19). HPV-positivity is defined by the detection of both HPV16 viral genomic DNA [measured with the Multiplex HPV Genotyping kit (Multimetrix, Heidelberg, Germany)] and HPV16 E6/E7 transcript (measured by quantitative RT-PCR) as described in (18).

### 2.2 Data sets and bioinformatic analyses

#### 2.2.1 Transcriptomics analysis

R and Bioconductor were used for the analysis of all data sets. The expression data obtained from microarrays Affymetrix HG-U133 plus 2.0 were obtained from HPV+ samples. Unsupervised analysis: A hierarchical classification allowed the observation of the distribution of samples. Ward’s aggregation criterion and correlation distance were used. The Principal Component Analysis was realized with the R FactomineR (V1.29) package (20).

#### 2.2.2 Differential analysis

The transcriptomic differential analysis was evaluated with a moderate t-test (R package limma V3.18.13) (21). The Bonferroni method was used for multiple corrections. Genes with a log fold change > 1 and an adjusted p-value< 0.05 were selected as differentially expressed. Results are presented as a heatmap (function heatmap, R package stats V3.0.2. R Core Team (2020). R: A language and environment for statistical computing. R Foundation for Statistical Computing, Vienna, Austria, https://www.R-project.org/.).

#### 2.2.3 Meta-analysis

Public data sets were acquired from the public database Gene Expression Omnibus (GEO) where only the HPV+ samples extracted from the upper aerodigestive tract (oropharynx, hypopharynx, larynx and buccal cavity) were selected. Based on these criteria, three data sets were obtained. Two of these data sets come from GEO Database, Slebos et al. (22) (GSE3292) and Pyeon et al. (23) (GSE6791). The last one was from a collaboration. The data sets were preprocessed to ensure that they are comparable, and clusters of highly interconnected genes were found in the reference data set using the WCGNA R package (V1.70-3) (24, 25) based on an unsupervised analysis of gene expression profiles. Next, we investigated how these clusters are conserved in the three other data sets using the Preservation function module of the WGCNA package. This function outputs a Z-score which indicates high (>10), moderate (5<Z<10) or lower preservation (Z<5) of the modules in other data sets. Finally, we determined which genes are highly connected in both sets.

#### 2.2.4 Analysis of the correlation between ΔNp63 and genes of signature identified by Barbieri et al. 

Expression of Barbieri’s signature genes (26) was analyzed in our data set. Correlation matrix of gene expression of these genes was calculated with the R package corrplot V0.73 and the function corrplot.

#### 2.2.5 Estimation of tissue-infiltrating immune cell population

The R package MCPcounter (V1.2.0) (27) was used, based on the normalized gene expression matrix.

### 2.3 Cell lines and reagents

The SCC90 cell line was a kind gift from Prof. Susan Gollin (University of Pittsburgh). SCC90 cells originate from a HPV16-positive oropharyngeal (base of tongue) tumor, and express wild-type *TP53* (28). The SCC47 cell line was a kind gift from Prof. Thomas E. Carey (University of Michigan). SCC47 cells originate from an HPV16-positive carcinoma of the tongue, and express wild-type *TP53* (29). Cells were maintained at 37°C with 5% CO_2_ and 90% humidity in Dulbecco’s modified Eagle’s medium (DMEM; PAN Biotech), supplemented with 10% fetal calf serum (FCS; Gibco), 10nM 4-(2-hydroxyethyl)-1-piperazineethanesulfonic acid (HEPES; PAN Biotech) and 1% non-essential amino acids (PAN Biotech). The THP-1 cell line was a kind gift from Elisabeth Martin (UMR1113, Strasbourg), and were maintained at 37°C with 5% CO_2_ and 90% humidity in Roswell Park Memorial Institute (RPMI) medium supplemented with 10% fetal bovine serum (Gibco).

### 2.4 siRNA and expression plasmid transfection

ΔNp63 and DKK3 downregulation was achieved by transfecting 15 nM of ΔNp63 siRNA (Eurogentec; sense strand: 5’-ACGAGGAGCCGTTCTAATC-3’; antisense strand: 5’-ACCTGGAAAACAATGCCCAGA-3’) and 15 nM of DKK3 siRNA (Santa cruz Biotechnology; sc-41102) to 3E^05^ SCC90 cells using Lipofectamine RNAiMAX, according to the manufacturer’s instructions. The Eurogentec SR-CL011-005 scrambled and Santa Cruz sc-37007siRNAs was used as a negative control for downregulation of ΔNp63 and DKK3, respectively. Total protein and RNA was extracted 48 h (DKK3) and 72 h (ΔNp63) post-transfection to assess gene and protein expression. CKAP4 downregulation was achieved by transfecting 15 nM of CKAP4 siRNA (Santa Cruz Biotechnology; sc-95758) to 5E^05^ THP-1 cells using Lipofectamine RNAiMAX, according to the manufacturer’s instructions. Santa Cruz scrambled siRNA (sc-37007) was used as a negative control.

ΔNp63 overexpression was achieved by transfecting 0.5 µg of pcDNA3-ΔNp63 vector to 5E^05^ SCC90 cells using the jetPRIME^®^ Polyplus-transfection reagent according to the manufacturer’s instructions. Empty pcDNA3 was used as a negative control. Total protein and RNA was extracted 48 h post-transfection to assess gene and protein expression.

### 2.5 Gene expression assays

Gene expression assays in tumor samples were performed by extracting total RNA from 77 OSCC frozen tissues using the RNeasy kit (Qiagen), according to the manufacturer’s instructions. The integrity of extracted RNA was verified on an Agilent 2100 Bioanalyser (Agilent Technologies). RNA was retro-transcribed using the Goscript reverse transcription system (Promega), and real-time quantitative PCR was performed using the LightCycler® 480 real-time PCR system (Roche). Specific primer pairs were used to measure the expression of the genes that encode ΔNp63, S100A9, THBS4, CD8α, GZMK and CD68 (see **Supplementary Table S2** for primer sequences). RT-qPCR data was analyzed using LightCycler® 480 software. The expression levels of each gene were normalized to the geometric mean Ct values of 2 internal controls, *Ribosomal Protein Long P0* (*RPL0*) and *Ubiquitin B* (*UBB*).

Gene expression assays on cultured cells were performed by extracting total RNA from pelleted cells using a standard TRIZol procedure (TRI Reagent®: TR 118 Molecular Research Center), according to the manufacturer’s instructions. RNA was retro-transcribed using the Goscript reverse transcription system (Promega), and real-time quantitative PCR was performed using the LightCycler® 480 real-time PCR system (Roche). *ΔNp63* and *DKK3* expression was measured with pairs of specific primers (see **Supplementary Table S2** for primer sequences) and normalized to the expression of *RPLP0* using the 2^-ΔΔCt^ method.

Total RNA harvested from THP-1 cells was retro-transcribed using the High-Capacity cDNA Reverse Transcription Kits (Applied Biosystems), and real-time quantitative PCR was performed using the Applied Biosystems QuantStudio3. cDNA was diluted five times before being used as a template according to the provider instructions (4 µL/reaction) with FastStart Universal Probe Master Mix Taqman. Specific primers are used from TaqMan Gene Expression Assay (Applied Biosystems) to measure the expression of *CXCL10* (Hs00171042_m1), *CCL4* (Hs99999148_m1), *IL1B* (Hs00174097_m1) and normalized to the expression of *ACTB* (Hs01060665_g1) using the 2^-ΔΔCt^ method.

### 2.6 SDS-PAGE and western blot analysis

Total protein extraction was carried out by homogenizing 10E^06^ cells in 100 µL of 1X Laemmli lysis buffer [6.25 mM Tris (pH 6.8), 1% SDS, 1% DTT, protease and phosphatase inhibitors, Sigma]. 20 or 40 μg of total proteins were resolved by 6%-15% SDS-PAGE (depending on protein molecular weight) according to standard methods. Proteins were detected with primary antibodies raised against p63, p53, HPV16 E6, cleaved caspase 3 (Cas3*), DKK3, IKBα, IKBα-pS32, AKT, AKT-pS473, CKAP4 (see **Supplementary Table S3** for clones, providers and concentrations). Depending on the host species, blots were probed with secondary antibodies (1/8000 anti-mouse IgG-HRP linked antibody, Cell Signalling 7076S; 1/8000 anti-rabbit IgG-HRP linked antibody, Cell Signalling 7074S). Proteins were visualized with enhanced chemiluminescence using the Clarity™ ECL Western blotting Substrate Bio-Rad reagent, according to the manufacturer instructions. Signals were acquired on a Pxi Imager (Syngene®) and quantified with the Genetools software (Syngene®).

### 2.7 Immunohistochemistry analysis and assessment

Immunohistochemical experiments were performed using a standard protocol extensively detailed previously (30, 31). The primary antibodies were as follows: anti-ΔNp63, anti-CD8, anti-CD68, anti-S100A7, anti-S100A9, anti-KRT6B and anti-THBS4 (see **Supplementary Table S3** for clones references and concentrations). The mouse or rabbit EnVision detection system (Dako) was used for the secondary reaction according to supplier’s recommendations. ΔNp63, S100A7, S100A9, KRT6B and THBS4 immunolabeled tissues were evaluated by histopathologists using a semi-quantitative score of the intensity (0: negative, 1: weak, 2: moderate, 3: intense) and extent (0:<5% positive cells, 1: 6–33%, 2: 34–66%, 3: >67%) of the staining, according to an arbitrary scale (30, 32). The multiplication of results acquired with the two scales allowed the obtention of a global score, ranging from 0 to 9. Tissue specimens with ΔNp63 scores below ≤4 and >4 were classified as ΔNp63^low^ and ΔNp63^high^, respectively. Regarding both CD8 and CD68 immunostainings, the number of positive cells per mm^2^ was determined by computerized counts (QuPath 0.2.0) (33).

### 2.8 Cell migration and cell invasion assays

The analysis of the migratory properties of SCC90 and SCC47 cells were performed using Boyden chambers. For this purpose, the cells are seeded in 6-well plates and then transfected 24 h later with either siRNA or expression vector (see above). The culture medium of the transfected cells was first replaced by FCS-free DMEM for 3 h. Then, cells were detached from their support with trypsin, counted, and 8E^04^ cells were seeded in Thincert Cell Culture Inserts (Greiner Bio One) in 500 μL of FCS-free medium. Inserts were placed in the wells of a 24-well plate containing 750 μL of DMEM with 10% FCS, and cultures were incubated for 24 h at 37°C with 5% CO_2_ and 90% humidity. After rinsing the inserts with 1X PBS, cells on the upper part of the insert were removed with a cotton-tipped swab, and cells that migrated through the filter to the lower part of the insert were fixed in 4% paraformaldehyde (PFA, Sigma-Aldrich) for 20 min at room temperature and stained with 1% crystal blue violet (Sigma). The filter of each chamber was then removed, mounted between slide and coverslip, and photographs were taken with a transmitted light microscope (Zeiss, Axio Imager.A1). The analysis of cell invasion was carried out in similar conditions, using matrigel-coated inserts (Corning® BioCoat® Matrigel®) and staining cells with a 1/50000 solution of DAPI (Sigma) for 20 min. In this case, photographs were taken with a Zeiss Axio Imager M2-Apotome2 fluorescence microscope.

### 2.9 *In vitro* cell viability analysis

#### 2.9.1 MTT cell viability assays

A total of 1E^04^ cells were seeded per well in 96–well microplates (Falcon Multiwell, Dutscher), 24 h prior to treatment. Cisplatin (Mylan) at different concentrations (0; 0.1; 0.5; 1; 2.5; 7.5; 15; 30; 100 μM) was applied for 48 h in 100 µL of fresh medium. MTT assay was performed as previously described by replacing the cisplatin solution with fresh medium supplemented with 5 mg/L MTT (Sigma) for 1 h (34). Cells were lysed in DMSO 100% (100 μL/well). Absorbance measurements were performed at 550 nm with the LB942 Tristar2 Multimode Reader (Berthold Technologies). The calculation of the IC_50_ and IC_75_ was performed with the GraphpadPrism V5.02 software (Graphpad, Software, USA) using non-linear regression.

#### 2.9.2 Resazurin (7-hydroxy-3H-phenoxazin-3-one) assays

SCC90 spheroids were obtained by seeding 5E^03^ cells in the wells of 96-well round-bottom microtest plates (Thermo Fisher Scientific), in 100 μL of culture medium. SCC90 cells were incubated for 24 h, and SCC47 cells were incubated for 48 h, at 37°C with 5% CO_2_ and 90% humidity to form spheres. SCC90 spheroids were further incubated with 11 μL of cisplatin solution at various concentrations (0; 0.1; 0.5; 1; 2.5; 7.5; 15; 30; 100 μM) and one volume of a 10% resazurin in DMEM medium, for 48 h at 37°C with 5% CO_2_ and 90% humidity. Absorbance measurements were performed at 550 nm with the LB942 Tristar2 Multimode Reader (Berthold Technologies).

### 2.10 RNA-sequencing and transcriptomic data analysis

RNA was extracted from SCC90 cells transfected with siRNA against ΔNp63 and TRIzol-mediated cell lysis. After extraction and RNA precipitation, supernatants were removed and the RNA pellet was washed with 75% EtOH, centrifuged at 9000× *g* for 5 min at 4°C, and again, 75% EtOH was added. Then, the RNA was resuspended in nuclease-free H_2_O and quantified using a NanoDrop Spectrophotometer (Thermo Scientific, Waltham, MA, USA). To identify the deregulated genes, RNASeq was performed on extracted total RNAs. RNA-Seq libraries were generated from 600 ng of total RNA using TruSeq Stranded mRNA Library Prep Kit and TruSeq RNA Single Indexes kits A and B (Illumina, San Diego, CA), according to manufacturer’s instructions. Briefly, following purification with poly-T oligo attached magnetic beads, the mRNA was fragmented using divalent cations at 94°C for 2 min. The cleaved RNA fragments were copied into first strand cDNA using reverse transcriptase and random primers. Strand specificity was achieved by replacing dTTP with dUTP during second strand cDNA synthesis using DNA Polymerase I and RNase H. Following addition of a single ‘A’ base and subsequent ligation of the adapter on double stranded cDNA fragments, the products were purified and enriched with PCR (30 sec at 98°C; [10 sec at 98°C, 30 sec at 60°C, 30 sec at 72°C] x 12 cycles; 5 min at 72°C) to create the cDNA library. Surplus PCR primers were further removed by purification using AMPure XP beads (Beckman-Coulter, Villepinte, France). The final libraries were checked for quality and quantified using capillary electrophoresis, and sequenced as single end read 1x50b on the Illumina HiSeq4000 platform according to manufacturer’s instructions. Image analysis and base calling were performed using RTA 2.7.3 and bcl2fastq 2.17.1.14. The quantification of gene expressions was performed with the Kallisto algorithm (v 0.46.2) and default options. RNA-Seq analysis was assessed with R software (R version 4.0.3). The differential analysis was performed with DESeq2 R package (version 1.30.0). A significantly differentially expressed gene corresponds to a p-value inferior to 0.005 and a log2 fold change ≥ 1. Pathways enrichment analyses were performed using multiple databases (*e.g*., DAVID, STRING, Reactome, TRAP, Biomarkers).

### 2.11 *In vitro* phagocytosis assay

SCC90 cells were seeded in 6-well plates and transfected with siΔNp63 (see above). Conditioned culture medium was harvested 48 h after transfection and filtered on 0.2 µm filters. THP-1 cells differentiation in macrophages was carried out by seeding 5E^05^ cells in wells of a 6-well microplate containing a coverslip, and by incubating them in a 162 nM PMA (phorbol 12-myristate 13-acetate; Sigma) solution in RPMI for 48 h. PMA solution was then replaced by fresh medium (70% RPMI; 30% SCC90 conditioned medium), and further incubated for 24 h. Seventy two hours after transfection and the initiation of THP-1 differentiation, SCC90 cells were incubated for 30 min with 1 mM of CellTRacker™ Green CMFDA Dye (Thermofisher) in FCS-free DMEM, and THP-1 cells were incubated for 30 min with 1 mM of CellTRacker™ Deep Red Dye (Thermofisher) in FCS-free RPMI. After medium replacement by FCS-complemented DMEM, SCC90 cells were gently scraped in 1X PBS, centrifuged for 5 min at 800 rpm, resuspended in DMEM, and seeded on THP-1 cells in a 1:1 cell ratio (one well of each culture was used for cell numeration). Co-cultures were maintained at 37°C for 4h. Culture medium was then removed, coverslips were washed in 1X PBS and THP-1 cells were fixed in 4% PFA for 10 min at room temperature, and nuclei were labeled with a DAPI solution (1/20000) for 5 min. Coverslips were mounted in Calbiochem FluorSave™ reagent (Merck Millipore), and pictures were taken with a Zeiss Axio Imager M2-Apotome2 fluorescence microscope. Finally, in order to confirm the phagocytosis activity, a real time imaging approach was used using IncuCyte^®^ S3 Live-Cell Analysis Instrument (SARTORIUS). THP-1 cells were seeded in a 6-well plate without coverslip and the phagocytosis experiment was carried out as described previously. Nine images per well from two technical replicates were taken every 10 min for 22 h using a 20X objective lens and then analyzed using the IncuCyte^®^ Basic Software. Green channel acquisition time was 400 ms, whereas red channel acquisition time was 800 ms.

### 2.12 Immunocytofluorescent staining assays

THP-1 cells differentiation in macrophages was carried out by seeding 5E^05^ cells in wells of a 6-well microplate containing a coverslip, and by incubating them in a 162 nM PMA (phorbol 12-myristate 13-acetate; Sigma) solution in RPMI for 48 h. PMA solution was then replaced by fresh medium and further incubated for 6 h with 0.5 µg of DKK3 recombinant protein (rhDKK3). Culture medium was then removed, coverslips were washed in 1X PBS and cells were fixed in 4% PFA for 10 min at room temperature. Permeabilization was achieved using 0.1% Triton X-100 in PBS for 5 min at 4°C and then blocked in 5%NGS/PBS for 30 min at room temperature. Coverslips were then soaked with NF-κB p65 antibody (1/400) during 60 to 90 min at room temperature and washed in 1X PBS for 15 min before and after incubation with secondary fluorescent antibody (Alexa Fluor™ 488 Rabbit, Invitrogen) for 30 min in darkness. Nuclei were labeled with a DAPI solution (1/20 000) for 5 min. Coverslips were mounted in Calbiochem FluorSave™ reagent (Merck Millipore), and pictures were taken with a Zeiss Axio Imager M2-Apotome2 fluorescence microscope.

### 2.13 Statistical and survival analyses

Survival analyses were performed using MedCalc statistical software (http://www.medcalc.be/). A two-sample Wilcoxon rank-sum (Mann-Whitney) test was used to evaluate the association between the gene expression level of *S100A9* and *THBS4* and the occurrence of metastatic spread at 3 years, and the Liu method (maximization of the product of sensitivity and specificity) (35) was used to determine optimal cut-off values. A prediction score was then constructed by entering THBS4 and S100A9 cut-off values in a logistic regression model. This score was used to stratify patients and evaluate the impact of this stratification on metastasis-free survival at 3 years using a univariate Kaplan-Meier survival analysis and log-rank post-test. A multivariate Cox regression model including the THBS4/S100A9 prediction score and potential confounding factors (age; gender; tumor stage; history of tobacco smoking) was used to evaluate their influence on metastasis-free survival.

The hypothesis of normality (d’Agostino and Pearson test; Shapiro-Wilk test) and homogeneity of variances (Levene test for equality of variances) of other data sets were analyzed. If the sample did not meet at least one of these conditions, then a non-parametric test was used (Wilcoxon-Mann-Whitney test). Otherwise, parametric tests were used (Student t-test; ANOVA and Bonferroni post-test; ANOVA and Tukey post-test). Statistical tests were performed using GraphPad Prism 8. For all analyses, significance is represented in graphs using asterisks: *p<0.05; **p<0.01; ***p<0.001; ****p<0.0001.

## 3 Results

### 3.1 HPV-positive OSCC display molecular and prognostic heterogeneity is linked to differential expression of ΔNp63

**Figure 1:**
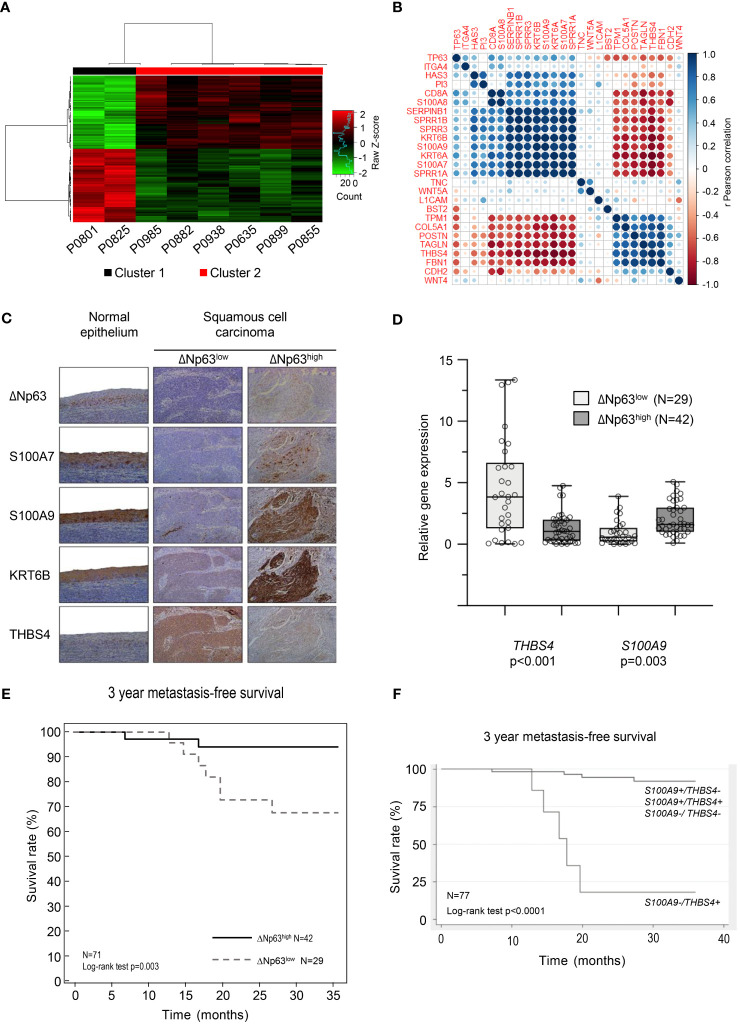
HPV-positive OSCC display molecular and prognostic heterogeneity that involves ΔNp63. **(A)** Dendograms and heat-map representation of unsupervised hierarchical Cluster 1 (poor prognosis) and Cluster 2 (good prognosis). **(B)** Correlation matrix of the expression of *TP63* and genes shown to be regulated by ΔNp63 by Barbieri et al. (26). **(C)** Immunohistochemistry analysis of the expression of ΔNp63, S100A7, S100A9, KRT6B and THBS4. **(D)** Box-and-whisker plot (minimum to maximum, showing all points) representation of the expression of the *THBS4* and *S100A9* genes in ΔNp63^low^ and ΔNp63^high^ HPV-positive OSCC. **(E)** Kaplan-Meier analysis of the 3-year metastasis-free survival of patients stratified according to the expression of the *THBS4* and *S100A9* genes. **(F)** Kaplan-Meier analysis of the 3-year metastasis-free survival of patients stratified according to the expression of the *S100A9* and *THBS4* genes.

We analyzed transcriptomic data from 8 tumor samples from patients with locally advanced HPV-16 positive OSCC. Unsupervised hierarchical clustering analysis distinguished two molecular groups (**Figure 1A**; Cluster 1 and Cluster 2), based on the differential expression of 148 genes (**Supplementary Table S4**). Interestingly, analysis of follow-up clinical data of the corresponding patients (**Table 1**) showed that Cluster 1 is associated with metastatic recurrence within 18 months and death within 3 years, and Cluster 2 with delayed metastatic spread (59 and 117 months after treatment) and longer survival (4/6 patients were still alive after 129 months). This shows that HPV-related OSCC can be separated into two clusters with different prognosis and molecular signatures.

**Table 1:** Demographics of patients in Cluster 1 and Cluster 2.

	Cluster 1		Cluster 2					
**Patient #**	P0801	P0825	P0985	P0882	P0938	P0635	P0899	P0855
**Gender**	Male	Male	Male	Male	Male	Male	Female	Female
**Age**	53	61	82	58	48	42	75	54
**Tobacco**	Yes	Yes	NA	No	Yes	Yes	No	Yes
**pT**	T3	T3	T2	T3	T2	T3	T2	T2
**pN**	N3	N2b	N2c	N2a	N1	N2b	N1	N2b
**Tumor stage**	IV	IV	IV	IV	III	IV	III	IV
**Treatment**	Surg.+RT	Surg.+RT	Surg.+RT	Surg.+RT	Surg.+RT	Surg.+RT	Surg.+RT	Surg.+RT
**Metastasis at 3 years**	Yes	Yes	No	No	No	No	No	No
**Metastasis-free survival**	17 months	20 months	59 months	174 months	117 months	170 months	173 months	135 months
**Overall survival**	35 months	20 months	60 months	174 months	129 months	170 months	173 Months	135 months
**5 years Overall survival**	Deceased	Deceased	Deceased	Alive	Alive	Alive	Alive	Alive

Two and six patients were found in Cluster 1 and Cluster 2, respectively. Patient number (#), gender and age, tobacco consumption, pathological tumor size staging (pT), pathological lymph node invasion staging (pN), tumor stage, treatment [Surgery (Surg.); Radiotherapy (RT)], metastasis at 3 years (yes vs. no), metastasis-free survival (in months), overall survival (in months) and the 5-year overall survival status (alive vs. deceased) are shown. NA, not available.

The significance of the clustering analysis was evaluated by comparisons with three independent HNSCC data sets from GEO [« Mirghani et al. » (E-MTAB-2146) (36), Slebos et al. (GSE3292) (22) and Pyeon et al. (GSE6791) (23)], using weighted correlation network analysis (WGCNA) (see flowchart in **Supplementary Figure S1A**). Clusters of highly interconnected genes (*i.e.*, modules), corresponding to gene co-expression networks, were identified in both the reference and comparison data sets using unsupervised hierarchical clustering and dynamic tree cut analysis (modules are indicated by colors in **Supplementary Figures S1B–G**; connected modules are assigned identical colors). The analysis was limited to probes that were expressed in the different data sets (*i.e*., probes expressed in our data set and in publicly available data sets). The degree of conservation of gene modules between each pairwise comparison was assessed using a Z-score, which was established for each matching cluster (color; **Supplementary Table S5**; a Z-score > 10 indicates high conservation while a Z-score ranging from 5 to 10 indicates moderate conservation). 25/100 genes in the “purple module” of Slebos et al. (asterisk in **Supplementary Figure S1B**; italicized Z-score in **Supplementary Table S5**), 23/100 genes in the “red module” of Mirghani et al. (asterisk in **Supplementary Figure S1D**; italicized Z-score in **Supplementary Table S5**), and 17/100 and 15/100 genes, respectively, in the midnight blue and pink modules of Pyeon et al. (asterisks in **Supplementary Figure S1F**; italicized Z-score in **Supplementary Table S5**) were also found to be downregulated in Cluster 2 (**Supplementary Table S4**; Gene names in bold). These observations, using three additional and independent transcriptomic data sets, support our initial finding of two transcriptomic profiles that correlate with distinct prognosis of HPV-positive OSCC patients.

Several genes that we found to be upregulated (*S100A7*, *S100A9*, *SPRR1A*, *SPRR1B*, *SPRR3*, *KRT6A*, *KRT6B*, *SERPINB1*; in bold in **Supplementary Table S4**) or downregulated (*THBS4*; in bold in **Supplementary Table S4**) in the good-prognosis Cluster 2 have been shown to be targets of the ΔNp63 transcription factor (26). We evaluated whether the expression of these targets and *TP63* are correlated in our data set. Correlation matrix analysis indicates that there is a correspondence (**Figure 1B**), suggesting that ΔNp63-dependent transcription could contribute to the different expression patterns of the two clusters. To evaluate if these RNA expression are also reflected at the protein level, we analyzed a separate collection of 71 formalin-fixed paraffin-embedded (FFPE) specimens from an independent cohort of HPV-positive OSCC patients (See **Supplementary Table S1** for patient demographics). Using semi-quantitative scoring of immunohistochemistry stains, tumors were stratified into two groups with high or low ΔNp63 expression (ΔNp63^high^ (N=42) and ΔNp63^low^ (N=29), respectively; **Figure 1C**). Consistent with our transcriptomic data, ΔNp63^high^ tumors expressed relatively high levels of S100A7, S100A9 and KRTB6 proteins (**Figure 1C**). RT-qPCR analysis of RNA harvested from this later collection of HPV-positive OSCC samples (fresh-frozen samples, N=77, **Supplementary Table S1**) showed that *S100A9* is more highly expressed in ΔNp63^high^, and *THBS4* in ΔNp63^low^ HPV-positive OSCC (**Figure 1D**).

We then evaluated whether gene expression levels are different in metastatic and non-metastatic tumors in this larger cohort (N=77). Using median expression and a two-sample Wilcoxon rank-sum test, we found that *S100A9*, *SERPINB1* and *SPRR1A* are significantly more highly expressed in tumors with no metastatic spread within 3 years (**Supplementary Table S6**), whereas the expression of *THBS4* is significantly lower (**Supplementary Table S6**).

In order to assess prognostic power and maximize sensitivity and specificity, cut-off expression values were determined for each genes (see **Supplementary Table S6** for detailed sensibility, specificity and area under the curve). An optimal predictor was found by combining the cut-off values of *THBS4* and *S100A9*. In a Kaplan-Meier analysis of 3-year metastasis-free survival, patients with low *S100A9* and high *THBS4* expression (*S100A9*-/*THBS4*+) were found to be at risk for metastatic progression (Log-rank test p-value<0.0001; **Figure 1E**). This predictor was found to be an independent prognostic marker in a Cox multivariate regression analysis that included potential confounding factors (including patient age and gender, tumor stage and history of tobacco smoking; **Table 2**). Interestingly, a Kaplan-Meier analysis of the 3-year metastasis-free survival of patients stratified according to ΔNp63 protein expression (**Figure 1C**) showed that ΔNp63^low^ HPV-positive OSCC are at higher risk for distant metastatic spread (log-rank test p-value=0.003; **Figure 1F**). Altogether, these observations show that ΔNp63 expression and a the *S100A9*/*THBS4* predictor define two distinct molecular and prognostic subgroups of HPV-related oropharyngeal tumors and suggest that ΔNp63 may play a role in tumor progression and response to therapy.

**Table 2:** Multivariate analyses of the prognostic value of the S100A9/THBS4 predictor for the 3-year metastasis-free survival by a Cox regression model that includes potential confounding factors (including patient age and gender, tumor stage and history of tobacco smoking).

	3-year metastasis-free survival		
	Hazard ratio	95% CI	p-value
**Predictor** S100A9-/THBS4+ (N=7) vs. others (N=70)	24.22	4.23-138.81	**<0.0001**
**Gender** Male (N=51) vs. Female (N=26)	0.91	0.14-5.73	0.920
**Age** ≤ 59 years old (N=35) vs. > 59 years old (N=42)	0.65	0.15-2.84	0.564
**Tumor stage** Stage III/IV (N=66) vs. Stage I/II (N=11)	3.27	0.34-31.68	0.306
**Tobacco** Current/former (N=47) vs. never smoker (N=29)	11.23	1.04-120.82	**0.046**

Factors that are significantly associated with prognostic are shown with a bold p-value.

### 3.2 ΔNp63 regulates migration and invasion of HPV-positive head and neck cancer cells

**Figure 2:**
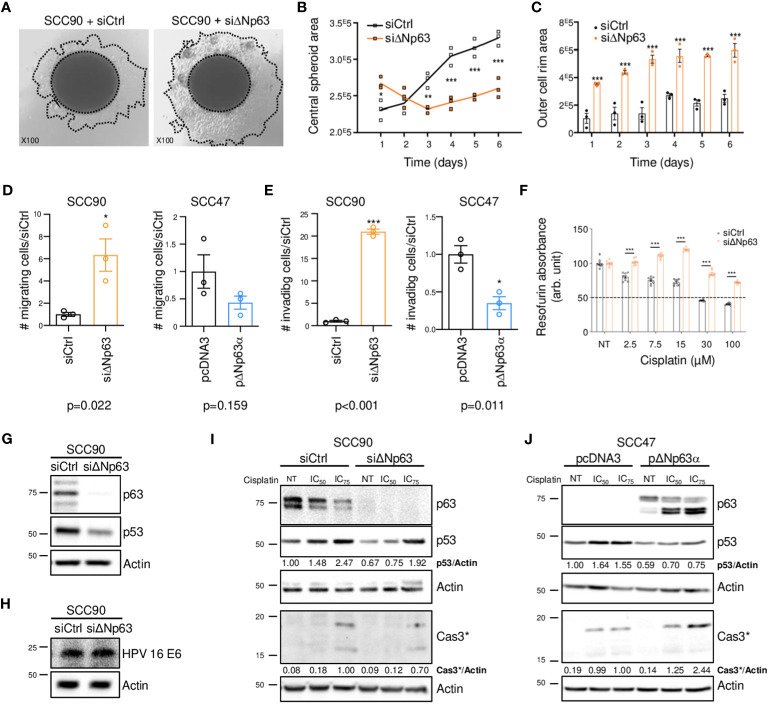
ΔNp63 regulates migration, invasion and response to cisplatin in HPV-positive head and neck cancer cells. **(A)** Spheroids of SCC90 cells transfected with scrambled or ΔNp63 siRNA. **(B)** Size of the central SCC90 spheroid area. Data is represented as individual replicates with mean connected (N=3). ANOVA and Bonferonni test: * p<0.05; ** p<0.01; *** p<0.001. **(C)** Size of the SCC90 spheroid outer cell rim area upon siRNA-mediated ΔNp63 downregulation. Data is represented as scatter plots with bars and mean +/- SEM (N=3). ANOVA and Bonferonni test: *** p<0.001. **(D)** Quantification of the migration of SCC90 and SCC47 cells upon ΔNp63 downregulation and upregulation, respectively (micrographs are shown in **Supplementary Figures S2E, H**). Data is represented as scatter plots with bars and mean +/- SEM (N≥3). Student t-test: * p<0.05. **(E)** Quantification of invasion of SCC90 and SCC47 cells upon ΔNp63 downregulation and upregulation, respectively (micrographs are shown in **Supplementary Figures S2E, H**). Data is represented as scatter plots with bars and mean +/- SEM (N≥3). Student t-test: * p<0.05. **(F)** Resazurin cell viability assay of SCC90 spheroids upon ΔNp63 inhibition. Data is represented as scatter plots with bars and mean +/- SEM (N=3). ANOVA and Bonferonni post-test: *** p<0.001. **(G, H)** Western blot analysis of ΔNp63, p53 **(G)** and HPV16 E6 **(H)** expression in siΔNp63-transfected SCC90 cells. **(I)** Western blot analysis of ΔNp63, p53 and cleaved caspase 3 (Cas3*) upon downregulation of ΔNp63 in SCC90 cells and treatment with cisplatin (IC_50_ = 2.8 μM; IC_75_ = 6.7 μM). p53 and Cas3* signals were quantified with respect to the actin loading control and normalized to non-treated siCtrl SCC90 cells or siCtrl SCC90 cells treated with the IC_75_ of cisplatin, respectively (quantification results are shown). **(J)** Western blot analysis of p63, p53 and cleaved caspase 3 (Cas3*) expression upon overexpression of ΔNp63 in SCC47 cells and treatment with cisplatin (IC_50_ = 2.7 μM; IC_75_ = 4.0 μM). p53 and Cas3* signals were quantified with respect to the actin loading control and normalized to non-treated siCtrl SCC90 cells or siCtrl SCC90 cells treated with the IC_75_ of cisplatin, respectively (quantification results are shown).

Since ΔNp63 expression is correlated with distant metastatic relapse (**Figure 1F**), we tested whether it might regulate cell migration and invasion. SCC90 cells, that express high levels of ΔNp63 have lower migration and invasion abilities compared to SCC47 cells, that express relatively low levels of ΔNp63 (**Supplementary Figures S2A, B**). Downregulation of ΔNp63 with a specific siRNA (siΔNp63; **Supplementary Figure S2C**) in sparse cultures of SCC90 cells resulted in greater cell flattening and coverage of the plate surface (**Supplementary Figure S2D**), and in the formation of smaller inner 3D spheroids (**Figures 2A, B**, especially days 3-6) with larger outer rims (**Figure 2C**). ΔNp63 silencing also resulted in increases in cell migration and invasion in transwell assays (**Supplementary Figure S2E**, **Figures 2D, E**, left graphs). Conversely, upregulation of ΔNp63 in SCC47 cells (**Supplementary Figure S2F**) resulted in changes in cell shape (**Supplementary Figure S2G**), and repression of cell migration (non-significant trend) and invasion (**Supplementary Figure S2H**, **Figures 2D, E**, right graphs). These observations show that ΔNp63 negatively regulates migration and invasion, and low levels of ΔNp63 are associated with increased migration and invasion in HPV-positive OSCC cells. This is consistent with increased risk for metastatic spread in tumors with low levels of ΔNp63 (ΔNp63^low^).

### 3.3 ΔNp63 is involved in the cell response to cisplatin and in cisplatin-induced cell apoptosis

To examine whether ΔNp63 expression affects carcinoma cell response to chemotherapy, we performed cell viability and spheroid assays using siRNA-transfected SCC90 cells treated with cisplatin. Decreased ΔNp63 expression led to decreased sensitivity to cisplatin in 2D-cultures (**Supplementary Figures S3A, B**). It also led to changes in spheroid morphology in 3D-cultures (**Supplementary Figure S3C**) as well as increased cell survival in 3D spheroids treated with increasing concentrations of cisplatin (**Figure 2F**). This shows that cells with lower ΔNp63 expression are more resistant to cisplatin-induced cytotoxicity. Downregulation of ΔNp63 in SCC90 cells decreases p53 protein expression (**Figure 2G**). This effect seems to be independent of HPV16 E6 oncoprotein, since E6 expression is not affected by ΔNp63 downregulation (**Figure 2H**). In addition, ΔNp63 knockdown decreases p53 induction by cisplatin (**Figure 2I**) as well as apoptosis (as measured by caspase 3 cleavage, **Figure 2I**). The effect on apoptosis appears to be mediated in part by p53, since p53 knockdown with p53 siRNA impaired, but did not completely abolish, caspase 3 cleavage (**Supplementary Figure S4**). Inversely, overexpression of ΔNp63 in SCC47 favored apoptosis induced by cisplatin (**Figure 2J**), which, however, did not correlate with increased expression of p53, suggesting that other factors could be involved. Overall, ΔNp63 expression levels affect apoptosis upon cisplatin treatment in cells in culture, suggesting that ΔNp63 levels affect sensitivity to platinum-based chemotherapy in HPV-positive carcinoma cells in human tumors.

### 3.4 ΔNp63 regulates the expression immune response genes and is linked to differences in the tumor immune landscape

**Figure 3:**
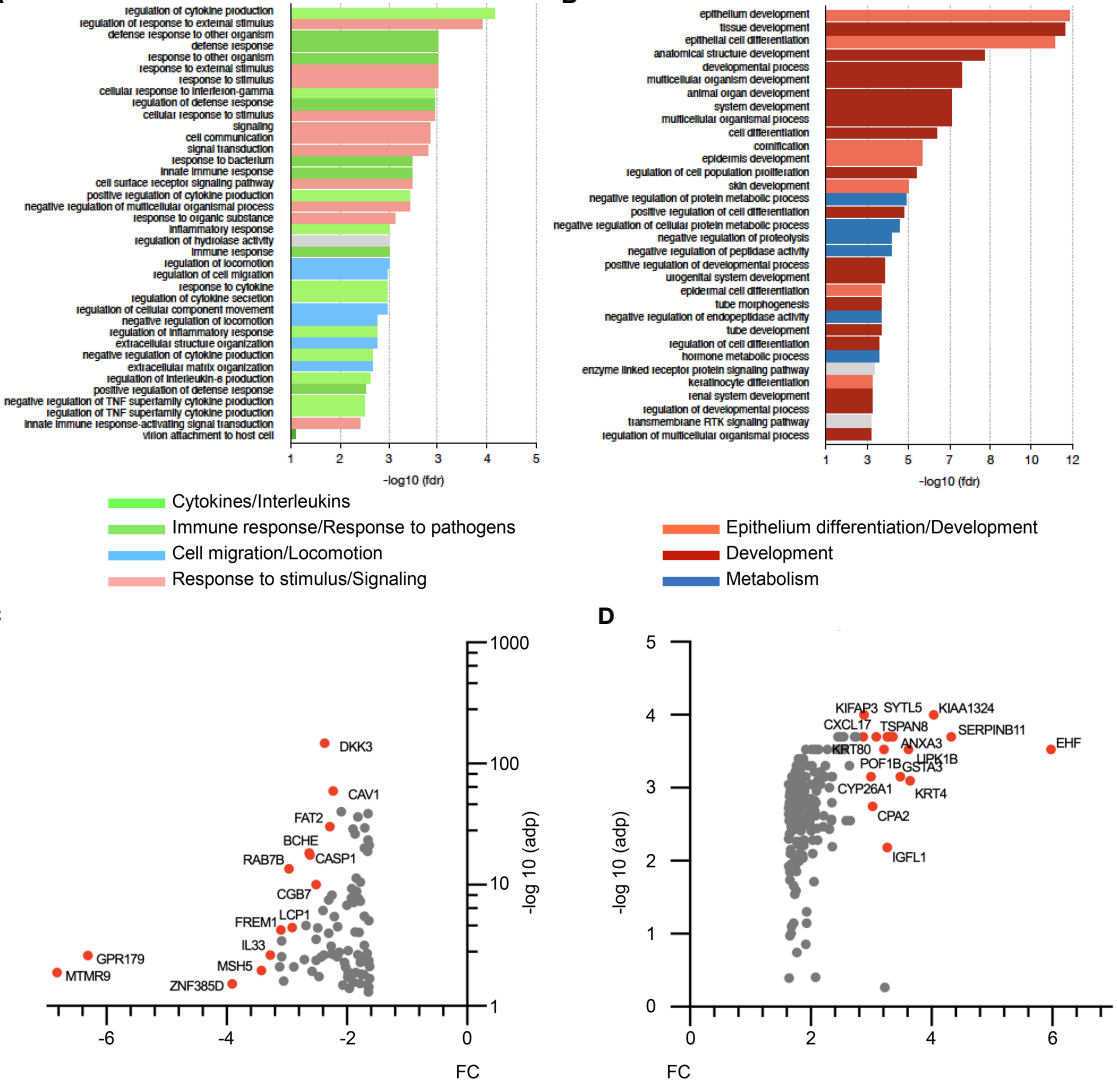
RNA-seq analysis of siΔNp63-transfected SCC90 cells. **(A)** Enrichment analysis of signalling pathways that are repressed upon siRNA-mediated ΔNp63 downregulation in SCC90 cells. **(B)** Enrichment analysis of signalling pathways that are activated upon siRNA-mediated ΔNp63 downregulation in SCC90 cells. **(C, D)** Top-100 downregulated **(C)** and upregulated **(D)** genes in siΔNp63-transfected SCC90 cells according to the fold change (FC) and the Log_10_.adjusted p-value [log_10_(adp)].

In order to gain further insights into ΔNp63-dependent mechanisms in HPV-positive OSCC, we investigated the transcriptional program regulated by ΔNp63 in SCC90 cells. RNA-sequencing (RNA-seq) analysis was carried out on cells transfected with either scrambled or ΔNp63 siRNA. Using a log_2_(fold change)>1 and adjusted p-value<0.05 as cut-offs, 690 genes were found to be differentially expressed (293 genes were downregulated and 397 upregulated) upon ΔNp63 inhibition. Pathway enrichment analysis using normalized transcriptomic data and the String software identified repressed (**Figure 3A**) and activated (**Figure 3B**) cellular functions. Most of the up-regulated genes were found to be involved in either development, epithelium/skin differentiation or cornification (**Figure 3B**; red and pink bars), as expected given the known role of ΔNp63 in keratinocyte differentiation and skin epithelium stratification. Consistent with the role of ΔNp63 in cell migration and invasion (**Figures 2A–F**), genes involved in movement and locomotion, as well as in the organization of the extracellular matrix, were significantly downregulated (**Figure 3A**; blue bars). More unexpectedly, the predominant downregulated pathways concern genes involved in the immune response and cytokine signalling (**Figure 3A**; light and dark green bars). The most downregulated genes (**Figure 3C**) include *RAB7B* (cellular response to interferon- γ; negative regulation of Toll-like receptor signalling; positive regulation of NF-κB signalling), *LCP1* (Interleukin-12-mediated signalling; T cell activation), *IL*-*33* (interleukin-33) and *DKK3* [inhibition of Wnt-signalling, interference with interferon-γ signalling and modulation of CD4+ and CD8+ T cell responses (37)]. Furthermore, one of the most upregulated genes is *CXCL-17* (involvement in monocyte, dendritic cell and macrophage chemotaxis; **Figure 3D**).

**Figure 4:**
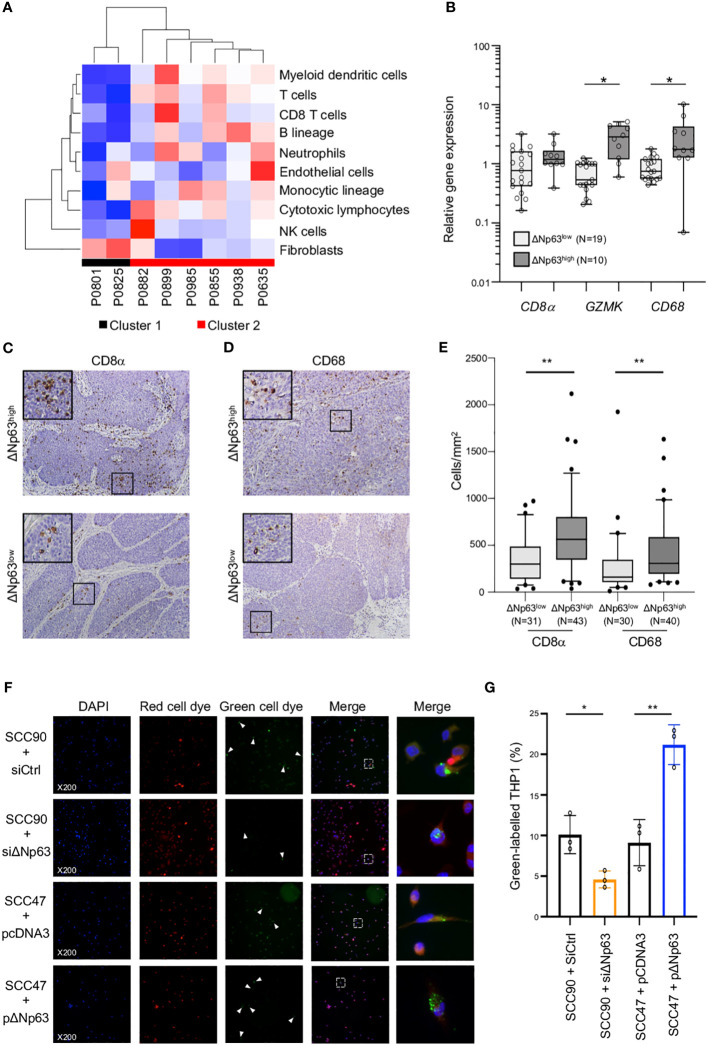
ΔNp63 is involved in the regulation of the immune response. **(A)** Deconvolution of the transcriptomic data (**Figure 1A**) and analysis of the abundance of 10 cell types in the tumor microenvironment of HPV-positive OSCC from Cluster 1 and Cluster 2. **(B)** Analysis of the expression of the CD8α, GZMK and CD68 genes in HPV-positive OSCC stratified according ΔNp63 levels (ΔNp63^low^ *vs*. ΔNp63^high^). Data is plotted as box plot (Box-and-whisker minimum to maximum, showing all points). ANOVA and Tukey post-test: * p<0.05. **(C, D)** Immunohistochemistry analysis of the expression of the CD8α **(C)** and CD68 **(D)** markers in formalin-fixed paraffin-embedded tumor samples of HPV-positive OSCC stratified according ΔNp63 expression. **(E)** Quantification of the number of CD8-positive T lymphocytes and CD68 macrophages (expressed as cells/mm^2^) in whole slides from HPV-positive OSCC stratified according ΔNp63 expression. Data is plotted as Box-and-whisker plots with the median and 25^th^ and 75^th^ percentile. Mann-Whitney two-tailed test: ** p<0.01. **(F)** Analysis of the *in vitro* phagocytosis of green-labeled SCC90 or SCC47) cells by red-labeled THP-1 macrophages upon ΔNp63 inhibition (siΔNp63) or overexpression (pΔNp63α). DAPI, Red cell dye, Green cell dye and merge are shown. White arrowheads indicate green-labeled phagocytosis vesicles. Magnification: X200. A magnification (right panels) of the inset in the merge is shown. **(G)** Quantification of the proportion (%) of THP-1 macrophages that display green-labeled phagocytosis vesicles. Data is represented as scatter plots with bars and mean +/- SEM (N=3). Two-tailed Student t-test: * p<0.05; ** p<0.01.

The clinical relevance of these observations was analyzed by correlating the expression of ΔNp63 and immune cell markers in HPV-positive OSCC samples. Immune-cell abundance in our initial cohort of 8 OSCC samples was analyzed by the MCP-counter method, which identifies immune cells based on specific gene signatures. Cluster 2 is clearly different from Cluster 1, and predicted to be enriched in myeloid dendritic cells, T cells, neutrophils, B and monocytic cell lineages and cytotoxic lymphocytes (**Figure 4A**). In order to confirm these observation, we quantified the expression of immune cell markers, including *CD8 α* (for CD8 T cells), *Granzyme K* (*GZMK*; for cytotoxic lymphocytes) and *CD68* (for macrophages). RT-qPCR analysis with RNA harvested from ΔNp63^high^ (N=10) *vs*. ΔNp63^low^ (N=19) HPV-positive fresh frozen samples showed that *GZMK* and *CD68* expression is higher in ΔNp63^high^ tumors (**Figure 4B**; ANOVA and Tukey post-test: * p<0.05). In addition, immunohistochemistry staining and automated signal quantification of CD8 α (**Figure 4C**) and CD68 (**Figure 4D**) in FFPE samples from ΔNp63^high^ and ΔNp63^low^ HPV-positive OSCC (N=+/-70) demonstrated that ΔNp63^high^ OSCC (N=43) had a significantly higher number of CD8 α -positive T cells (641 cells/mm^2^ versus 347 cells/mm^2^) compared to ΔNp63^low^ tumors (N=31) (**Figure 4E**; Mann-Whitney two-tailed p=0.0013). Similarly, ΔNp63^high^ OSCC (N=40) had an elevated number of CD68-positive macrophages (455 cells/mm^2^versus 293 cells/mm^2^) compared to ΔNp63^low^ tumors (N=30) (**Figure 4E**; Mann-Whitney two-tailed p=0.0051). These results provide compelling evidence for the contribution of the immune microenvironment to the prognosis of HPV-linked OSCC.

### 3.5 ΔNp63 regulates the uptake of HPV-positive head and neck cancer cells by macrophages

To explore the functional regulation of the immune response by ΔNp63, we carried out *in vitro* phagocytosis assays with THP-1-derived macrophages. THP-1 cells were stimulated with phorbol 12-myristate 13-acetate (PMA), labeled with a red fluorescent cell tracker dye and briefly co-cultivated (4 h) with SCC90 cells labeled with a green fluorescent cell tracker dye. ΔNp63 silencing in SCC90 cells decreased the percentage of THP-1 macrophages with green-labeled phagocytosis vesicles (**Figure 4F**) from 10.1% +/- 2.4 in siCtrl-SCC90 to 4.6% +/- 1.0 (**Figure 4G**; two tailed Student t-test p=0.021). Conversely, upregulation of ΔNp63 in SCC47 cells increased green label containing phagocytotic THP-1 macrophages (21.2% +/- 2.5), compared to mock-transfected SCC47 (9.1% +/- 2.8; **Figure 4B**; two tailed Student t-test p=0.005). Cancer cell phagocytosis was seen to occur by visualization with time-lapse video-microscopy (Video S1), which helps to rule out that the green vesicles were cell culture-related artifacts. These results show that ΔNp63 expression stimulates carcinoma-cell phagocytosis by macrophages.

### 3.6 ΔNp63 regulates cancer cell uptake by macrophages via DKK3 and the activation of a CKAP4-NF-κB axis

**Figure 5:**
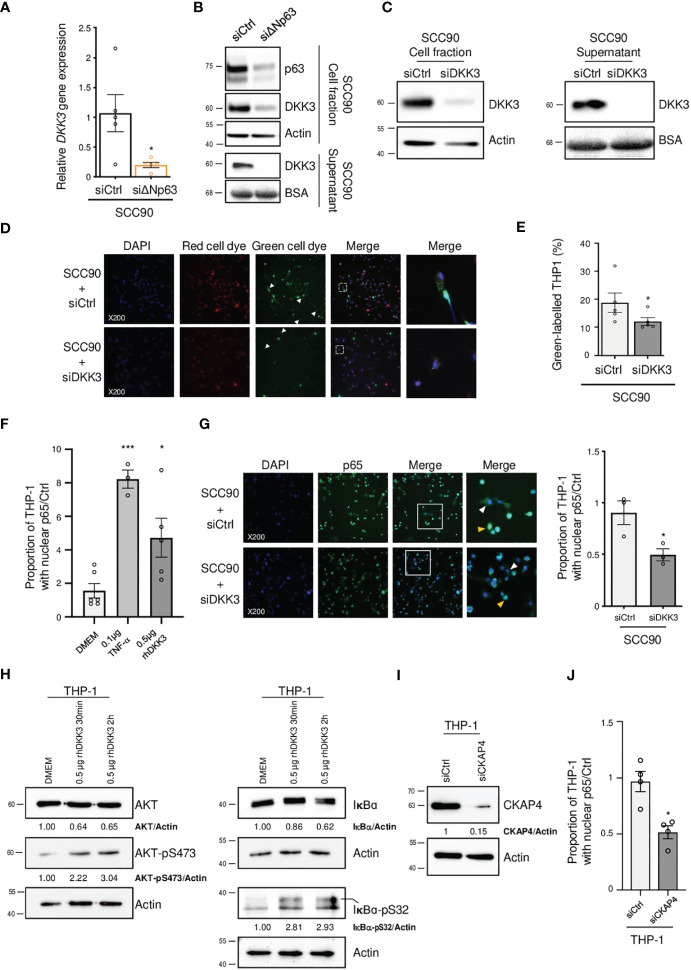
ΔNp63 regulates cancer cell uptake by macrophages via DKK3. **(A)** Analysis of the expression of the *DKK3* gene in siΔNp63-transfected SCC90 cells. Data is represented as scatter plots with bars and mean +/- SEM (N=4). Two-tailed Student t-test: * p<0.05. **(B)** Analysis of the expression of the DKK3 protein in the cellular (upper panels) and supernatant (lower panels) fractions of siΔNp63-transfected SCC90 cells. **(C)** Analysis of the expression of the DKK3 protein in the cellular (left panels) and supernatant (right panels) fractions of SCC90 cells transfected with an anti-DKK3 siRNA. **(D)** Analysis of the *in vitro* phagocytosis of green-labeled SCC90 cells by red-labeled THP-1 macrophages upon siRNA-mediated DKK3. DAPI, Red cell dye, Green cell dye and merge are shown. White arrowheads indicate green-labeled phagocytosis vesicles. Magnification: X200. A magnification (right panels) of the inset in the merge is shown. **(E)** Quantification of the proportion (%) of THP-1 macrophages that display green-labeled phagocytosis vesicles. Data is represented as scatter plots with bars and mean +/- SEM (N=4). Two-tailed Student t-test: p=0.029. **(F)** Quantification of the proportion of THP-1 macrophages that display p65-positive nuclei. THP-1 cells were incubated with DMEM (negative control), 0.1 µg of TNF-α or 0.5 µg of hrDKK4 for 6 h prior to staining (see also **Supplementary Figure S6A**). Data is represented as scatter plots with bars and mean +/- SEM (N≥3). ANOVA and Tukey post-test: *p<0.05; *** p<0.001. **(G)** Immunocytofluorescence analysis of the expression of p65 in THP-1 macrophages incubated with conditioned medium from siCtrl- (upper panels) or siDKK3- (lower panels) transfected SCC90 cells. DAPI, p65 staining and merge are shown. Magnification: X200. A magnification (right panels) of the inset in the merge is shown. White and yellow arrowheads highlight p65 staining in the cytoplasm and the nuclei, respectively. A quantification of the proportion of THP-1 macrophages with p65-positive nuclei is plotted in the graph. Data is represented as mean scatter plots with bars and +/- SEM (N=3). Two-tailed Student t-test: * p<0.05. **(H)** Western blot analysis of the expression of total and phosphorylated (pS473) AKT (left panels), and of total and phosphorylated (pS32) IκB-α in whole protein extracts from THP-1 cells incubated with rhDKK3 for 30 min and 2 h. AKT, AKT-pS473, IκB-α and IκB-α-pS32 signals were quantified with respect to the actin loading control and normalized to THP-1 macrophages incubated with DMEM (quantifications are shown). **(I)** Western blot analysis of CKAP4 expression in siCKAP4-transfected THP-1 macrophages. CKAP4 signals were quantified with respect to the actin loading control and normalized to siCtrl-transfected THP-1 macrophages (quantifications are shown). Shown blots are representative examples of three independent experiments. **(J)** A quantification of the proportion of siCtrl- and siCKAP4-transfected THP-1 macrophages with p65-positive nuclei upon incubation with 0.5 µg of rhDKK3 for 6 h. Data is represented as scatter plots with bars and mean +/- SEM (N=4). Two-tailed Student t-test: * p<0.05.

In order to study how ΔNp63 regulates the immune response, we investigated *DKK3*, which is one of the most significantly downregulated factors upon siRNA-mediated inhibition of ΔNp63 in SCC90 cells (**Figure 3C**; see above). We investigated this regulation further. We found that DKK3 expression depends on ΔNp63 in SCC90 cells at the transcriptional level (**Figure 5A**) and the protein level, both intracellularly (**Figure 5B**, upper panels) and extracellularly (**Figure 5B**, lower panels). These results suggest that ΔNp63 modulates DKK3 secretion by cancer cells, which could affect the properties of other cells, such as immune cells. We investigated whether DKK3 could stimulate THP-1 macrophages to phagocytose SCC90 cells, using *in vitro* phagocytosis assays. As a control, we showed that DKK3 siRNA transfection downregulated intra- and extra-cellular levels of the DKK3 protein (**Figure 5C**, left and right panels, respectively). THP-1 macrophages were cultivated for 24 h with conditioned medium from siRNA-transfected SCC90, and were further briefly co-cultivated with transfected green-labelled SCC90 (**Figure 5D**). Silencing of DKK3 significantly decreased green-label incorporation in THP-1 macrophage from 18.7% +/- 3.5 in siCtrl-SCC90 to 12.1% +/- 1.4 (**Figure 5E**; Mann Whitney two tailed p=0.028). Altogether, these results indicate that ΔNp63 regulates cancer cell phagocytosis by macrophages through DKK3 expression and secretion.

In order to decipher the molecular mechanisms of THP-1 macrophage activation by DKK3, we studied whether DKK3 affects the nuclear localization of ß-catenin, since DKK3 activity has been shown to negatively regulate ß-catenin and Wnt-signalling (38). THP-1 macrophages were incubated with recombinant human DKK3 (rhDKK3) protein and immunocytofluorescently stained for ß-catenin. We did not observe a statistically significant change in the number of ß-catenin-positive nuclei of THP-1 cells treated 6 h with 0.5 µg of rhDKK3, compared to the negative control (*i.e*., DMEM cell medium; **Supplementary Figure S5**), suggesting that nuclear localization of ß-catenin and Wnt signalling is not inhibited by rhDKK3. Consequently, we analyzed an alternative pathway, nuclear translocation of NF-κB, using p65 immunocytofluorescent staining. Interestingly, we found that incubation of THP-1 cells for 6 h with either Tumor Necrosis Factor-α (TNF-α; used as positive control) or 0.5 µg rhDKK3 triggered significant nuclear translocation of p65 (**Figure 5F**; **Supplementary Figure S6A**). As expected from activation of NF-κB, the expression levels of known NF-κB target genes, including *C-C Motif Chemokine Ligand 4* (*CCL4*), *C-X-C Motif Chemokine Ligand 10* (*CXCL10*), *Interleukin 1 beta* (*IL1B*) and *NF-Kappa-B Inhibitor Alpha* (IKBA), were found to be upregulated as early as 2 h after treatment with rhDKK3 (**Supplementary Figure S6B**). Strikingly, the proportion of THP-1 macrophages with p65-positive nuclei was found to be significantly lower (unpaired t-test p=0.034) after 6 h incubation with conditioned medium from siDKK3-transfected SCC90 cells (0.49+/-0.06; **Figure 5G**) compared to medium from siCtrl-SCC90 cells (0.90+/-0.12; **Figure 5G**). These observations show that extracellularly secreted DKK3 stimulates the NF-κB signalling pathway in THP-1 macrophages.

DKK3 was previously shown to bind to Cytoskeleton-Associated Protein 4 (CKAP4) on esophageal cancer cells (39), and DKK3 and CKAP4 were found to be required for the phosphorylation of the Akt serine/threonine kinase in HNSCC cell lines (40). In addition, Akt is known to be required for IκBα phosphorylation and degradation and subsequent NF-κB activation. We tested whether DKK3 has similar effects on THP-1 macrophages. Using western blot analysis of whole protein extracts from THP-1 macrophages, we observed that, as early as 30 min, DKK3 induced both Akt phosphorylation on serine S473, as well as IκBα phosphorylation on serine S32 (**Figure 5H**, left and right panels, respectively). Consistent with NF-κB nuclear translocation resulting from IκBα degradation, IκBα protein levels decreased following THP-1 incubation with DKK3. To further investigate the link between the CKAP4 receptor and NF-κB in THP-1 macrophages, we studied the consequences of CKAP4 receptor silencing on NF-κB nuclear translocation induced by DKK3. Using p65 immunocytofluorescent staining, we found that silencing of CKAP4 (**Figure 5I**) led to significantly fewer p65-positive nuclei in THP-1 cells following incubation with DKK3 for 6 h (**Figure 5J**, unpaired t-test p=0.02; **Supplementary Figure S7**). Altogether, these observations suggest that the expression of ΔNp63 in HPV-positive OSCC cells regulates NF-κB signalling in immune cells via the regulation of the expression of DKK3.

## 4 Discussion

### 4.1 ΔNp63 defines HPV-Positive OSCC molecular and prognostic heterogeneity

Multiple lines of evidence indicate that HPV-related OSCC are heterogeneous. Early on, Ang and collaborators (41) stratified HPV-positive patients into low and intermediate risk-of-death categories. Based on hierarchical clustering of transcriptomic data, HPV-positive HNSSC can be subdivided into two subgroups, HPV-classical (CL) and HPV-inflamed/mesenchymal (IMS) (9). The HPV-IMS subgroup has higher expression of mesenchymal-related and immune response genes and is associated with improved overall survival. However, the molecular mechanisms involved in causing this differential patient prognosis are still poorly understood. Similar to the previous report, our study also uncovers two molecular subgroups with distinct prognosis associated with distant metastasis. Importantly, our study finds that ΔNp63 makes a significant contribution to the different prognoses of the two groups. The ΔNp63 signature (ΔNp63-regulated genes) and high ΔNp63 protein levels were found to be biomarkers for good prognosis for HPV-related oropharyngeal cancer, the major site of HPV infection (42). Whether these findings are relevant for other sites of head and neck cancer remains to be determined.

There are rare instances of HPV infection in non-oropharyngeal head and neck locations. Interestingly, a recent meta-analysis found that HPV is associated with improved outcome in patients with laryngeal cancer (43). The contribution of ΔNp63 to the prognosis of these patients remains to be determined. HPV-related oropharyngeal cancer is mainly located in the lingual and palatine tonsils (42). In the remaining oral region, ΔNp63 expression in preneoplastic lesion has been reported to be associated with oral cancer risk (44). *TP53* mutations are rare in HPV-related OSCC but are frequently found in HPV-negative head and neck cancer (for review see (45)and references therein). The functional consequences of ΔNp63 expression in a mutant *TP53* background remains to be investigated.

The mechanisms that lead to differential expression of ΔNp63 in the two subgroups of HPV-positive tumors are not known, but could involve miR-203 expression or viral integration. Work by McKenna et al. suggests that the HPV16 E6 oncoprotein is indirectly involved in the regulation of miR-203 (46), and miR-203 regulates of the expression of ΔNp63 (47). The *TP63* genomic locus is a hotspot for HPV genome integration, resulting in genomic rearrangement and overexpression of local genes (48). These mechanisms that link HPV infection to the differential expression of ΔNp63 in tumors are interesting subjects for future studies.

We have found that high p63 is associated with good prognosis in OSCC. However, this is not always the case. p63 has a complex role in cancer, and can be oncogenic or tumor suppressive, depending on the cancer. For instance, high ΔNp63 expression correlates with poor patient outcome, tumor progression and/or metastasis in various cancers (49, 50). In contrast, low ΔNp63 expression correlates with high cancer progression in others (51, 52). An intriguing question is what determines these differences in ΔNp63’s contribution to tumor progression.

### 4.2 ΔNp63 is involved in cell migration/invasion and response to cisplatin

Higher expression of ΔNp63 in HPV-positive OSCC cells favors apoptosis induced by cisplatin and reduces cell migration/invasion, which could account for differences in prognosis. Interestingly, similar observations on cell migration and invasion were recently reported for HPV-positive squamous cells of the uterine cervix (53). In addition, and similarly to our finding, ΔNp63 expression levels in HNSCC tumors have been correlated with response to chemotherapy: patients with complete or partial tumor response were reported to have 4-6-fold higher ΔNp63 levels compared to non-responders (54). An important question is how ΔNp63 stimulates the cisplatin response? A possibility might be that ΔNp63 could increase access of cisplatin to DNA by remodeling inaccessible chromatin regions as proposed by Yu et al. (55). Our results also indicate that the mechanisms involve p53, since silencing of ΔNp63 in SCC90 cells reduces p53 protein levels, which are associated with DNA damage caused by cisplatin (56). It seems that regulation of HPV E6 protein is not involved in this process. Intriguingly, the overexpression of ΔNp63 in SCC47 cells also resulted in p53 expression downregulation, rather than an upregulation, suggesting that other factors might be involved. This complex balance of the interplay between p53, p63 and p73 and of their respective TA and ΔN isoforms has been reported to be delicate, and to be tissue dependent (57). A more in-depth study is required to decipher the molecular mechanisms involving ΔNp63 expression modulation and their effect on p53 expression.

### 4.3 ΔNp63 regulates the expression of diffusible immunomodulators that control cancer cell phagocytosis via a DKK3/CKAP4/NF-κB-dependent mechanisms

Our results provide, to the best of our knowledge, the first demonstration that ΔNp63 expression correlates with lymphocyte infiltration of HPV-related OSCC and that ΔNp63 regulates macrophage activity. These effects on infiltration and macrophages could contribute to differences in HPV-positive OSCC prognosis. Furthermore, we dissected the underlying mechanisms and show that ΔNp63 exerts this activity via the expression of diffusible factors, notably DKK3, which induces a CKAP4/NF-κB pathway in macrophages. This is consistent with previous studies that suggest that ΔNp63 participates in the regulation of chemokines or cytokines, including CCL17 (58), CXCL2 and CCL2 (49). There is some understanding of how ΔNp63 regulates immune response genes: ΔNp63 physically interacts with the c-Rel transcription factor (59, 60), thereby regulating inflammatory and immune response genes, as well as NF-κB (61). Our results also show that ΔNp63 expression functionally activates HPV-positive OSCC cell phagocytosis by macrophages, and that this mechanism appears to involve DKK3. DKK3 is an atypical member of a family of glycoproteins (DKK1-4) that act as inhibitors of the Wnt/ß-catenin signalling pathway (62, 63). This pathway plays an important role in the differentiation and biological activity of macrophages (64). DKK3 is secreted into the extracellular space by mesenchymal stem cells, where it modulates peripheral T-cell tolerance (37, 65). Previous studies propose that DKK3 has a tumor-suppressor role, through its ability to induce dendritic cell differentiation and activation of T cells (66, 67). In this report, we have identified additional ΔNp63 regulated genes, such as IL-33 or LCP1, that should be investigated further for their contribution to immune modulation by ΔNp63.

### 4.4 DKK3 stimulates the canonical NF-κB signalling pathway through its CKAP4 receptor

We show here that, in THP-1 macrophages, DKK3 induces the phosphorylation of IκBα on the S32 residue, IκBα protein level downregulation, p65 nuclear translocation and the upregulation of known NF-κB targets genes. These observations indicate that DKK3 activates the canonical NF-κB signalling pathway (68), which is known to regulate the phagocytic activity of macrophages (69). NF-κB has previously been shown to upregulate *CCL4*, *IL1BI*, *IKBA* and *CXCL10* genes in response to lipopolysaccharide stimulation of THP-1 macrophages (70). Interestingly, CXCL10 secretion by monocytes/macrophages is known to play a chemotactic function on T lymphocytes (71). Intriguingly, we observed that ΔNp63^high^ OSCC have a higher infiltration of both CD68 macrophages and CD8 T lymphocytes. Our *in vitro* results and our observations in patient tumors suggest that ΔNp63 regulates immunostimulatory diffusible factors that could activate macrophages that could in turn contribute to the recruitment of T lymphocytes. However, how our *in vitro* observations reflect the situation in human tumors remains yet to be determined.

The only known receptor of DKK3 is the CKAP4 type II transmembrane protein, which binds all DKK family proteins (72, 73). Both DKK1 and DKK3 have been reported to activate the PI3K/Akt signalling pathway and to trigger Akt phosphorylation via binding to CKAP4 (40, 74). This is consistent with our results, which show that Akt is phosphorylated in THP-1 cells upon incubation with rhDKK3. Interestingly, Akt is known to activate the NF-κB-pathway via the phosphorylation of IκB kinase-α (IKKα) (75). In addition, DKK1 has been shown to induce the activation of NF-κB signalling via the CKAP4 receptor in multiple myeloid cells. We propose that a DKK3/CKAP4/NF-κB axis is responsible for the activation of THP-1 macrophages. Our results implicate the canonical NF-κB signalling in the effects of DKK3 on THP-1 cells, but we cannot exclude that additional molecular mechanisms are also involved.

This study establishes that in HPV-positive OSCC, the elevated expression of ΔNp63 in cancer cells favors the recruitment of anti-tumor immune cells, and more specifically the activation of macrophages via the transactivation and secretion of DKK3 that in turn binds to CKAP4 on macrophage membranes and induces the NF-κB pathway.

## 5 Conclusions

Our results demonstrate that ΔNp63 is a tumor suppressor in HPV-positive OSCC, and that it favors response to therapy and infiltration of immune cells, while reducing migration and invasion. The mechanisms involve intercellular communication mediated by peptides and proteins that are localized in membranes or secreted, such as DKK3. These peptides and proteins constitute an “exteriome” that functions exterior to cells. Components of the exteriome could potentially be very useful for the stratification of HPV-positive OSCC, since they are readily detectable. Serum DKK3 is an emerging diagnostic biomarker for colorectal (76) and some gynecological cancers (77). In ovarian cancers, lower DKK3 levels are associated with an increased risk of cancer and lymphatic metastasis (77). In addition, we found that the expression at the RNA level of *THBS4* together with *S100A9* is an independent prognostic signature that remains predictive of metastasis despite confounding factors that include patient age, tobacco consumption and tumor stage. We propose that DKK3, as well as *THBS4* + *S100A9*, could be used in addition to conventional clinical features, to identify patients who are eligible for therapeutic de-escalation.

## Data availability statement

The datasets presented in this study can be found in online repositories. The names of the repository/repositories and accession number(s) can be found below: https://www.ebi.ac.uk/arrayexpress/, E-MTAB-1328 https://www.ncbi.nlm.nih.gov/geo/, GSE190046.

## Ethics statement

The studies involving humans were approved by Comité de protection des personnes Est IV. The studies were conducted in accordance with the local legislation and institutional requirements. The participants provided their written informed consent to participate in this study. Ethical approval was not required for the studies on animals in accordance with the local legislation and institutional requirements because only commercially available established cell lines were used.

## Author contributions

JM: Formal Analysis, Visualization, Writing – original draft, Investigation, Validation. CL: Formal Analysis, Investigation, Validation, Visualization, Writing – review & editing. AN: Formal Analysis, Investigation, Visualization, Writing – review & editing. JD: Investigation, Writing – review & editing. CBou: Investigation, Validation, Writing – review & editing. CM: Investigation, Validation, Writing – review & editing. PR: Investigation, Writing – review & editing. SL: Writing – review & editing, Resources. PS: Writing – review & editing. CBor: Writing – review & editing. MB: Writing – review & editing. BW: Writing – review & editing, Funding acquisition. GM: Writing – review & editing. MH: Writing – review & editing, Conceptualization, Investigation. CG: Conceptualization, Investigation, Writing – review & editing, Formal Analysis, Funding acquisition, Project administration, Supervision, Visualization. AJ: Conceptualization, Formal Analysis, Funding acquisition, Project administration, Supervision, Visualization, Writing – original draft.
