# Supplementary figures and images for: A novel ΔNp63-dependent immune mechanism improves prognosis of HPV-related head and neck cancer
Source: Front Immunol. 2023 Oct 25;14:1264093. doi: 10.3389/fimmu.2023.1264093 (PMC10630910; doi:10.3389/fimmu.2023.1264093)

Figure S1

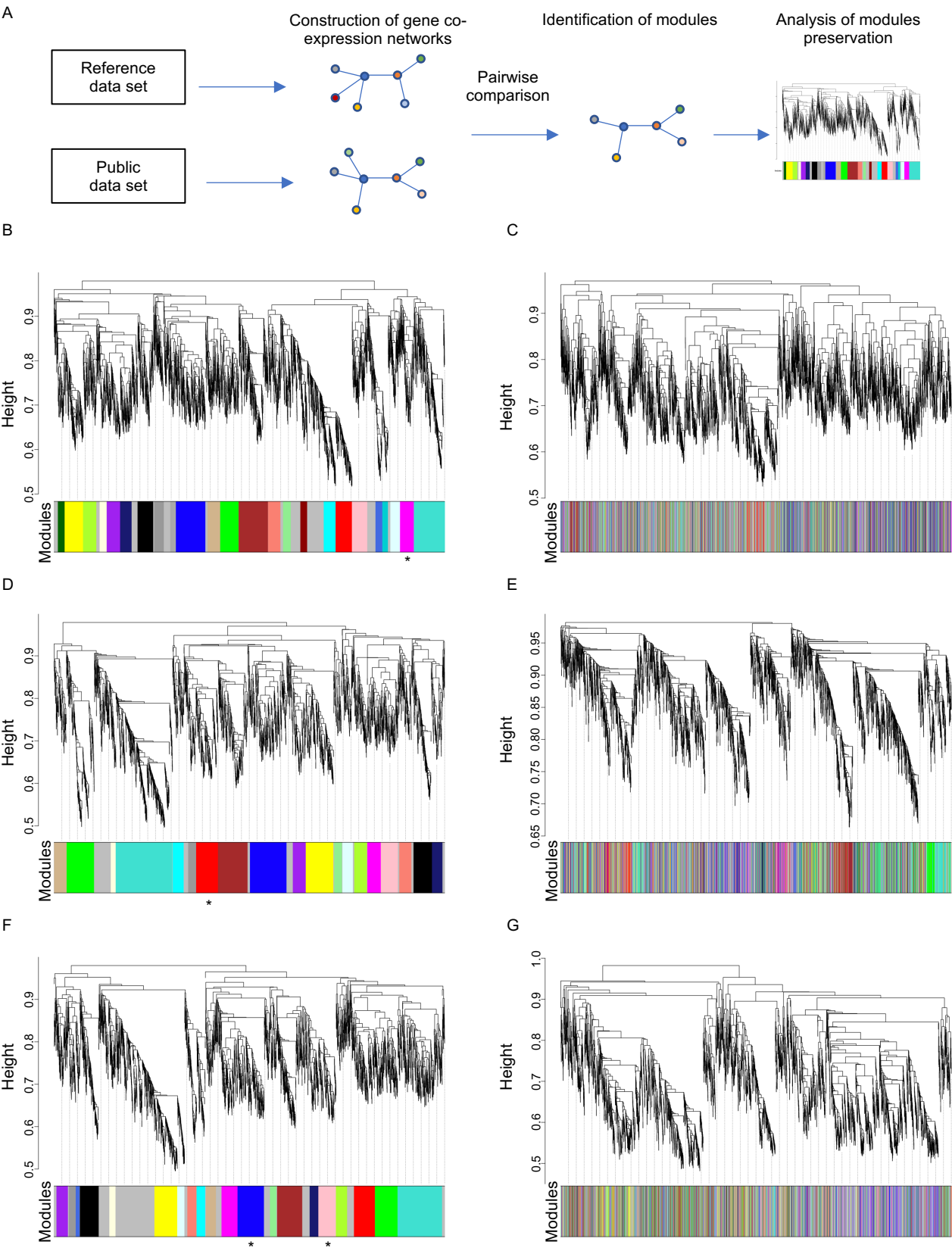

Figure S2

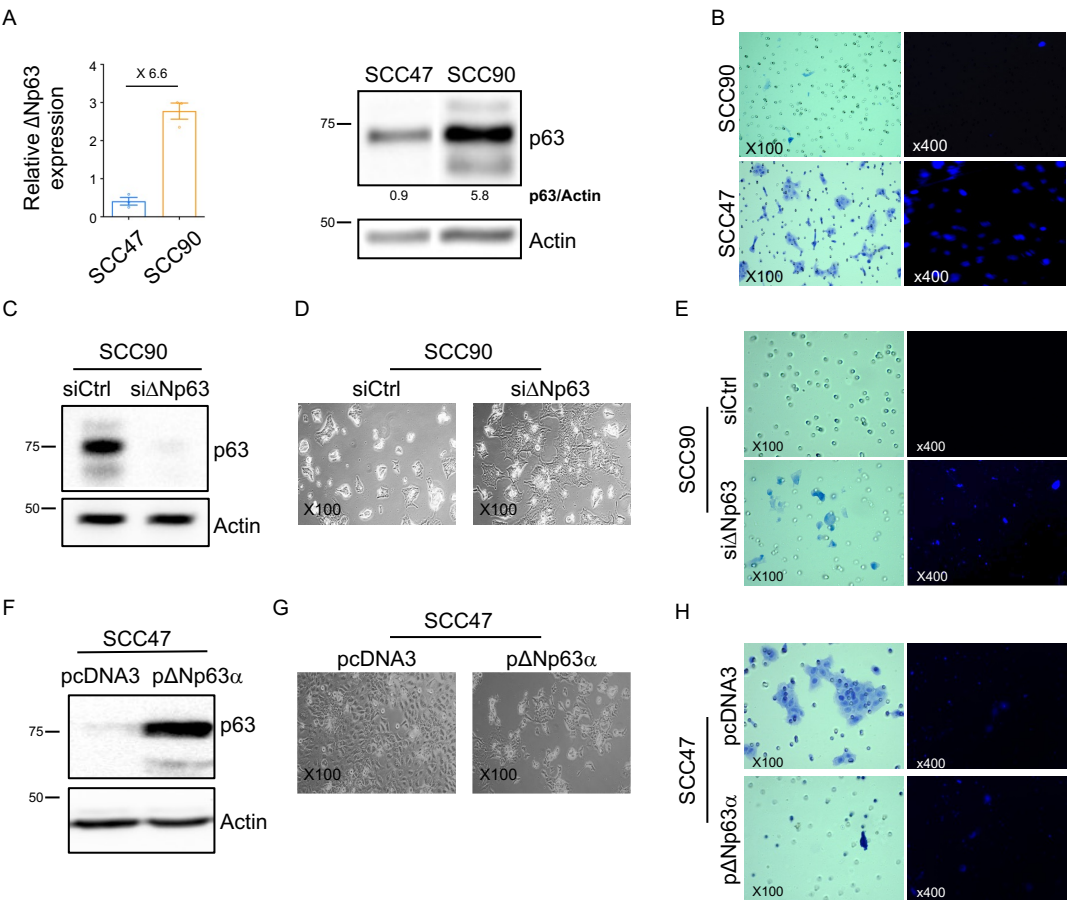

Figure S3

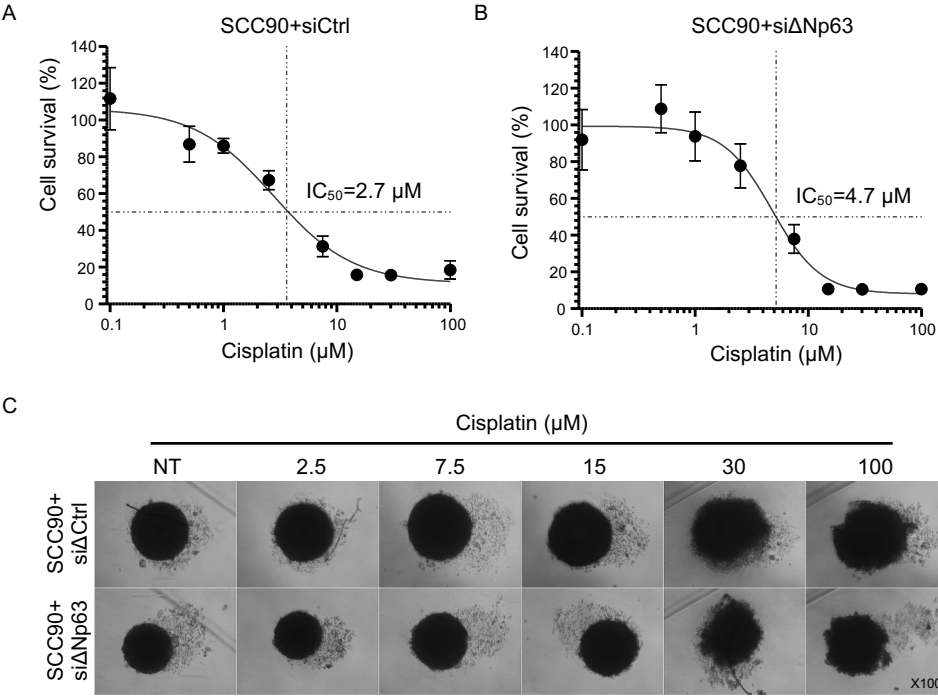

Figure S4

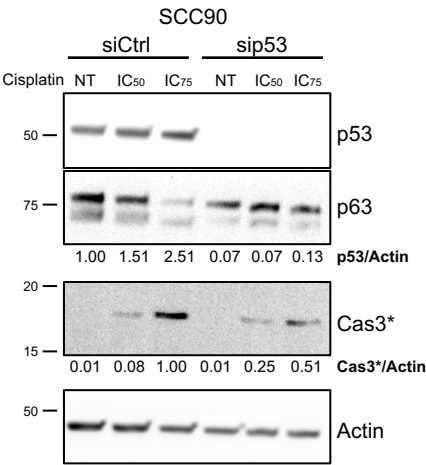

Figure S5

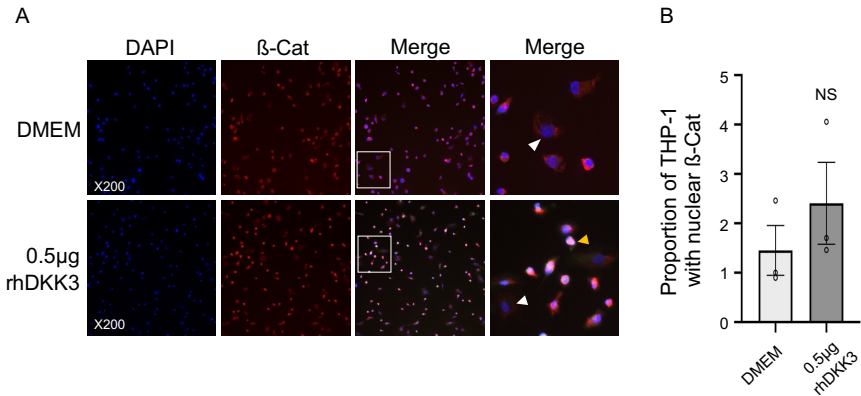

Figure S6

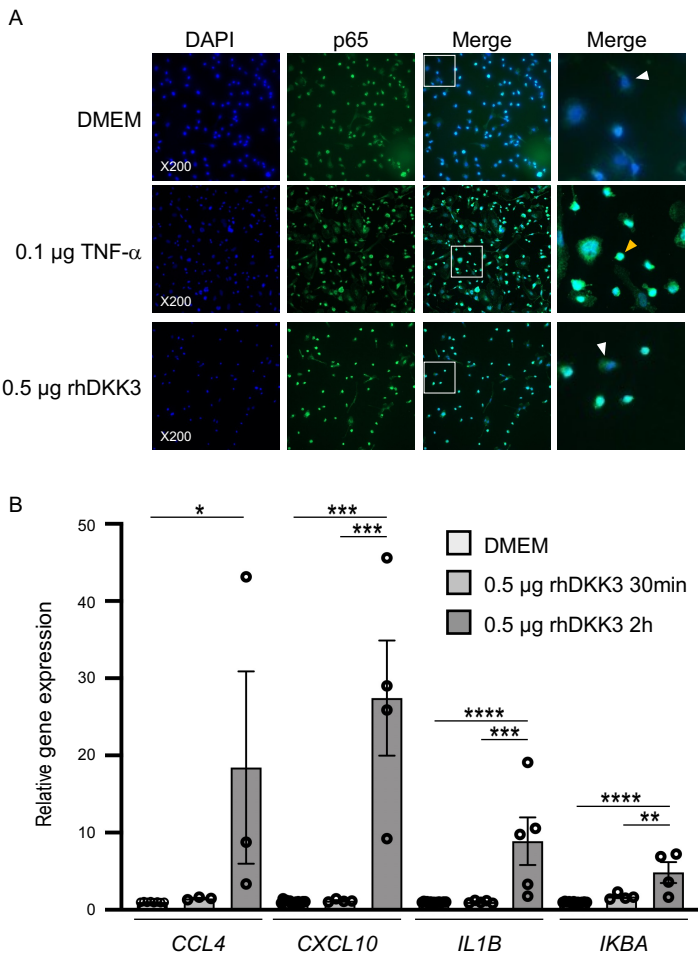

Figure S7

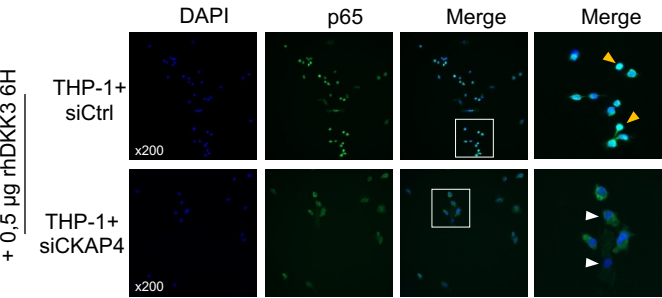

Supplement: Supplementary file 1 [file DataSheet_1.pdf]
