# Supplementary material for: A novel ΔNp63-dependent immune mechanism improves prognosis of HPV-related head and neck cancer
Source: Front Immunol. 2023 Oct 25;14:1264093. doi: 10.3389/fimmu.2023.1264093 (PMC10630910; doi:10.3389/fimmu.2023.1264093)
Supplement: Supplementary file 2 [file DataSheet_2.pdf]

Supplementary Material

Article Title A novel ΔNp63-dependent immune mechanism improves prognosis of HPV-related head and neck Cancer

Jana MOURTADA, Christelle LONY, Anaïs NICOL, Justine DE AZEVEDO, Cyril BOUR, Christine MACABRE, Patrick RONCARATI, Sonia LEDRAPPIER, Philippe SCHULTZ, Christian BOREL, Mickaël BURGY, Bohdan WASYLYK, Georg MELLITZER, Michaël HERFS, Christian GAIDDON*, Alain C. JUNG*

*** Correspondence:**

Dr Alain JUNG, Laboratoire de Biologie Tumorale, Institut de Cancérologie Strasbourg Europe, 17 rue Albert Calmette, F.67200 Strasbourg, FRANCE. Tel: +33 (0)3 88 27 53 67. Fax: +33 (0)3 88 26 35 38.
a.jung@icans.eu
Dr Christian GAIDDON, IRFAC INSERM U1113, 3 avenue Molière, F.67200 Strasbourg, FRANCE. Tel: +33 (0)3 88 27 53 67. Fax: +33 (0)3 88 26 35 38.
gaiddon@unistra.fr

1 Supplementary Tables

Supplementary Table 1: Patients' demographics. Two independent cohorts of patients with HPV-positive OSCC from the tumor banks of Strasbourg, France (N=34) and Liège, Belgium (N=43) were used for the validation of the transcriptomic data. 77 tumors specimens were used for gene expression assays by RT-qPCR. 71 FFPE samples were available for immunohistochemistry analyses. Patients' gender and age, history of tobacco smoking, pathological tumor size staging (pT), pathological lymph node invasion staging (pN), tumor stage, treatment, occurrence of metastasis within three years after treatment and 5-year overall survival are shown. Number and percentage (in brackets) for each cohort and combined cohorts are shown. NA: non available.

	**HPV-positive OSCC Strasbourg (N=34)**	**HPV-positive OSCC Liège (N=43)**	**HPV-positive OSCC Total (N=77)**
**Gender**			
Male	21 (62%)	30 (70%)	51 (66%)
Female	13 (38%)	13 (30%)	26 (34%)
**Age**			
Age<60 years	16 (47%)	19 (44%)	35 (45%)
Age≥60 years	18 (53%)	24 (56%)	42 (55%)
**History of tobacco smoking**			
Never smoker	9 (26%)	20 (47%)	29 (38%)
Former/current smoker	24 (71%)	23 (53%)	47 (61%)
NA	1 (3%)	0 (0%)	1 (1%)
**Pathological tumor size staging (pT)**			
T1	5 (15%)	5 (11.5%)	10 (13%)
T2	16 (47%)	27 (63%)	43 (56%)
T3	12 (35%)	6 (14%)	18 (23%)
T4	1 (3%)	5 (11.5%)	6 (8%)
**Pathological lymph node staging (pN)**			
N0	6 (18%)	9 (21%)	15 (19%)
N1	7 (20%)	12 (28%)	19 (25%)
N2a	4 (12%)	3 (7%)	7 (9%)
N2b	11 (32%)	14 (32.5%)	25 (32%)
N2c	4 (12%)	5 (11.5%)	9 (12%)
N3	2 (6%)	0 (0%)	2 (3%)
**Tumor stage**			
Stage I	1 (3%)	3 (7%)	4 (5%)
Stage II	1 (3%)	6 (14%)	7 (9%)
Stage III	11 (32%)	12 (28%)	23 (30%)
Stage IV	21 (62%)	22 (51%)	43 (56%)
**Treatment**			
Surgery	2 (6%)	8 (18.5%)	10 (13%)
Surgery + Radiotherapy	19 (56%)	14 (32.5%)	33 (43%)
Surgery + Chemoradiotherapy	13 (38%)	21 (49%)	34 (44%)
**Metastasis at 3 years**			
Yes	4 (12%)	5 (11.5%)	9 (12%)
No	29 (88%)	38 (88.5%)	67 (88%)
**Overall survival at 5 years**			
Alive	27 (80%)	30 (70%)	57 (84%)
Deceased	7 (20%)	13 (30%)	20 (26%)

Supplementary Table 2: List of oligonucleotide primer pairs. Gene of interest expression analysis was carried out by using a RT-qPCR approach. The names of analyzed gene are shown, as well as the 5'-to-3' sequence of forward and reverse oligonucleotide primers used in this study.

**Gene name**	**Forward primer (5'-3')**	**Reverse primer (5'-3')**
ΔNp63	ACGAGGAGCCGTTCTGAATC	ACCTGGAAAACAATGCCCAG
S100A7	GCCTGCTGACGATGATGAAG	ATGGCTCTGCTTGTGGTAGT
S100A9	GGACCTGGACACAAATGCAG	CTGTGATCTTGGCCACTGTG
KRT6B	TCTAGGTCCAGCTGCAGATG	GAGAGCAGAGAAAGCAGTGC
SERPINB1	AAGTTTGGCTCTGTTGGCTG	TCCCATGGCTATCAGGAGGA
SPRR1A	AGTTAGCATGCTGTCACCCT	CATCCTCAAATGCACCCGAG
SPRR1B	CTCTTCACACCAGGACCAGT	GCTCCTTGGTTTTGGGGATG
THBS4	GCAGACAGAGATGGCATTGG	ATCGGTGTCTTTCTGGTCGT
CD8a	AGGAAGTGAACCTGGTGGTG	CTCAGCAGACACTGCCACAT
GZMK	GTATTTTGGCAGGACCAGGA	CATTCCTGTGGGCTTTTTGT
CD68	ACTGAACCCCAACAAAACCA	TTGTACTCCACCGCCATGTA
DKK3	TTCATCCAGCAGTGTTGCTC	GGTGTGGGGTAGTGGAGAGA
RPLP0	GAAGGCTGTGGTGCTGATGG	CCGGATATGAGGCAGCAGTT
UBB	GCTTTGTTGGGTGAGCTTGT	CGAAGATCTGCATTTTGACCT
IKBα	CAGCAGACTCCACTCCACTT	GAGAGGGGTATTTCCTCGAA
ACTB	ATTGCCGACAGGATGCAGAA	GCTGATCCACATCTGCTGGAA

Supplementary Table 3: List of antibodies and experimental conditions. Protein of interest expression analysis was carried out by using Western Blot, immunohistochemistry or immunocytofluorescence approaches. Protein names are shown, as well as the references of used antibodies (including clone reference and provider) and the dilution at which they were used is shown.

	**Protein**	**Provider**	**Antibody dilution**
**Western blot**	Actine	mab150, Millipore	1/15000
	p63	4A4 ab-735, Abcam	1/500
	p53	DO-1 sc-126, santa Cruz	1/1000
	HPV16 E6	E6-6F4, Euromedex	1/500
	Cleaved caspase 3	9661L, Cell signaling	1/1000
	DKK3	ab-186409, Abcam	1/1000
	BSA	B2901, Sigma	1/2000
	anti-mouse IgG-HRP linked antibody	7076S, Cell Signaling	1/8000
	anti-rabbit IgG-HRP linked antibody	7074S, Cell Signaling	1/8000
	CKAP4	PA5-51455, Invitrogen	1/2500
	IKBα	14D4, Cell signaling	1/1000
	IKBα-Ps32	44D4, Cell signaling	1/1000
	AKT	92725, Cell signaling	1/1000
	AKT-pS473	4060L, Cell signaling	1/2000
**Immunohistochemistry**	p63	BC28 ab-172731, Abcam	1/150
	S100A7	HPA006997, Sigma-Aldrich	1/500
	S100A9	HPA004193, Sigma-Aldrich	1/10000
	KRT6B	17391-1-AP, Thermo Fisher Scientific	1/800

	THBS4	G-10, Santa Cruz Biotechnology	1/100
	CD8	SP57, Roche Tissue Diagnostics	1/1
	CD68	KP-1, Roche Tissue Diagnostics	1/1
**Immunocytofluoresence**	Anti-rabbit IgG Alexa Fluor 488	Invitrogen	1/1000
	NFκB P65	sc-372, Santa Cruz	1/400

Supplementary Table 4: List of 148 genes found to be differentially expressed in Cluster 1 and Cluster 2. Gene symbols, Gene names, gene id, log_2 fold-change (logFC), average expression (AveExpr) and adjusted p-value (Adj.p.Val) are shown. Please note that only genes found to be deregulated with a logFC>1 and an Adj.p.Val<0.05 in our unsupervised hierarchical clustering analysis (Fig. 1A) are shown. Gene names highlighted in bold were previously shown to be transcriptionally regulated by ΔNp63 in Barbieri *et al.* (1).

**Gene symbol**	**Gene name**	**gene id**	**logFC (>1)**	**AveExpr**	**Adj.p.Val (< 0,05)**
**S100A7**	S100 calcium binding protein A7	6278	-8,12	9,73	5,54E-03
**SPRR1A**	small proline-rich protein 1A	6698	-6,89	10,19	2,94E-04
**SPRR1B**	small proline-rich protein 1B	6699	-6,32	10,18	5,15E-03
**SPRR1A**	small proline-rich protein 1A	6698	-6,14	9,90	4,96E-03
CLCA4	chloride channel accessory 4	22802	-6,13	7,48	4,54E-02
**SPRR3**	small proline-rich protein 3	6707	-5,44	10,80	1,32E-02
TMPRSS11E	transmembrane protease, serine 11E	28983	-5,17	7,10	2,05E-02
C10orf99	chromosome 10 open reading frame 99	387695	-5,03	6,89	4,98E-02
CLCA2	chloride channel accessory 2	9635	-5,02	7,08	2,86E-02
SBSN	suprabasin	374897	-4,90	7,63	4,98E-02
CRNN	cornulin	49860	-4,79	7,64	4,78E-02
CLCA2	chloride channel accessory 2	9635	-4,75	7,69	3,47E-02
IVL	involucrin	3713	-4,73	7,65	4,96E-03
SPINK5	serine peptidase inhibitor, Kazal type 5	11005	-4,45	8,88	4,02E-02
FAM83C	family with sequence similarity 83, member C	128876	-4,41	6,26	4,65E-02
CLCA2	chloride channel accessory 2	9635	-4,29	6,97	4,59E-02
RHCG	Rh family, C glycoprotein	51458	-4,21	8,02	4,78E-02
GBP6	guanylate binding protein family, member 6	163351	-4,21	6,72	2,77E-02
GJB6	gap junction protein, beta 6, 30kDa	10804	-4,19	7,01	2,62E-02
**KRT6B**	keratin 6B	3854	-4,18	11,21	8,39E-03
**SPRR3**	small proline-rich protein 3	6707	-4,16	11,77	1,48E-02
SPRR2C	small proline-rich protein 2C (pseudogene)	6702	-4,14	5,38	2,70E-02
TGM3	transglutaminase 3	7053	-4,08	6,66	8,39E-03
TGM1	transglutaminase 1	7051	-3,90	7,15	3,26E-02
CYP2E1	cytochrome P450, family 2, subfamily E, polypeptide 1	1571	-3,75	6,19	2,76E-02
**KRT6A**	keratin 6A	3853	-3,40	11,21	8,80E-03
**S100A9**	S100 calcium binding protein A9	6280	-3,36	11,61	4,35E-02

CCR7	chemokine (C-C motif) receptor 7	1236	-3,21	5,51	4,43E-02
IL1RN	interleukin 1 receptor antagonist	3557	-3,20	8,27	4,37E-02
IL1RN	interleukin 1 receptor antagonist	3557	-3,14	9,44	4,35E-02
**KRT6B**	keratin 6B	3854	-3,02	11,22	2,62E-02
SELL	selectin L	6402	-2,97	6,92	3,48E-02
DSG3	desmoglein 3	1830	-2,95	9,15	2,00E-02
GRHL3	grainyhead-like 3 (Drosophila)	57822	-2,89	5,87	4,35E-02
ZNF750	zinc finger protein 750	79755	-2,72	5,37	4,83E-02
GJB5	gap junction protein, beta 5, 31.1kDa	2709	-2,69	6,52	4,02E-02
DSG3	desmoglein 3	1830	-2,67	9,28	4,35E-02
NLRC3	NLR family, CARD domain containing 3	197358	-2,65	5,06	3,47E-02
TTC22	tetratricopeptide repeat domain 22	55001	-2,43	7,10	2,86E-02
ZBED2	zinc finger, BED-type containing 2	79413	-2,31	5,12	4,98E-02
ANKRD22	ankyrin repeat domain 22	118932	-2,26	7,76	1,63E-02
**SERPINB1**	serpin peptidase inhibitor, clade B (ovalbumin), member 1	1992	-2,21	8,02	1,32E-02
**SERPINB1**	serpin peptidase inhibitor, clade B (ovalbumin), member 1	1992	-2,13	8,89	2,62E-02
ANKRD22	ankyrin repeat domain 22	118932	-2,09	6,77	4,65E-02
PRKCB	protein kinase C, beta	5579	-2,07	5,44	4,98E-02
LCK	LCK proto-oncogene, Src family tyrosine kinase	3932	-2,06	7,10	3,48E-02
HLA-DOB	major histocompatibility complex, class II, DO beta	3112	-2,03	5,54	4,02E-02
ANXA1	annexin A1	301	-1,99	11,50	4,81E-02
GJB3	gap junction protein, beta 3, 31kDa	2707	-1,96	7,55	1,13E-02
DAPP1	dual adaptor of phosphotyrosine and 3-phosphoinositides	27071	-1,95	5,32	3,99E-02
IL2RB	interleukin 2 receptor, beta	3560	-1,91	7,48	2,15E-02
ITGAL	integrin, alpha L (antigen CD11A (p180), lymphocyte function-associated antigen 1; alpha polypeptide)	3683	-1,86	6,11	3,47E-02
EPHA1	EPH receptor A1	2041	-1,81	5,85	2,70E-02
LCK	LCK proto-oncogene, Src family tyrosine kinase	3932	-1,80	6,04	4,59E-02
ERO1L	ERO1-like (S. cerevisiae)	30001	-1,78	7,23	4,59E-02
GJB3	gap junction protein, beta 3, 31kDa	2707	-1,76	7,14	2,62E-02
SEPT1	septin 1	1731	-1,75	5,13	2,45E-02
ARHGAP15	Rho GTPase activating protein 15	55843	-1,70	6,81	4,78E-02
DAPP1	dual adaptor of phosphotyrosine and 3-phosphoinositides	27071	-1,61	6,02	4,83E-02
PTPRC	protein tyrosine phosphatase, receptor type, C	5788	-1,60	8,57	4,78E-02
EVI2B	ecotropic viral integration site 2B	2124	-1,50	7,20	4,98E-02
DUSP28	dual specificity phosphatase 28	285193	-1,50	5,18	5,00E-02
SPN	sialophorin	6693	-1,44	4,22	4,54E-02
MAP4K1	mitogen-activated protein kinase kinase kinase kinase 1	11184	-1,40	5,18	3,47E-02

RHOF	ras homolog family member F (in filopodia)	54509	-1,38	4,11	4,78E-02
ZNF101	zinc finger protein 101	94039	-1,38	4,66	4,65E-02
LINC00324	long intergenic non-protein coding RNA 324	284029	-1,33	4,16	3,87E-02
FYB	FYN binding protein	2533	-1,32	7,65	4,78E-02
HMGCR	3-hydroxy-3-methylglutaryl-CoA reductase	3156	-1,25	6,60	4,65E-02
ZNF200	zinc finger protein 200	7752	-1,22	5,76	4,35E-02
GAS7	growth arrest-specific 7	8522	1,13	3,76	4,78E-02
GABRB2	gamma-aminobutyric acid (GABA) A receptor, beta 2	2561	1,21	2,30	3,47E-02
CALD1	caldesmon 1	800	1,26	2,81	4,78E-02
DDX43	DEAD (Asp-Glu-Ala-Asp) box polypeptide 43	55510	1,39	2,78	4,78E-02
ZNF853	zinc finger protein 853	54753	1,39	3,44	4,35E-02
LAMA4	laminin, alpha 4	3910	1,44	3,60	4,78E-02
SSPN	sarcospan	8082	1,44	4,41	4,43E-02
SGCD	sarcoglycan, delta (35kDa dystrophin-associated glycoprotein)	6444	1,46	3,17	4,37E-02
MFGE8	milk fat globule-EGF factor 8 protein	4240	1,47	6,68	4,59E-02
PCDHB16	protocadherin beta 16	57717	1,48	2,74	4,96E-02
TANC1	tetratricopeptide repeat, ankyrin repeat and coiled-coil containing 1	85461	1,51	3,32	4,37E-02
MAGI2-AS3	MAGI2 antisense RNA 3	1E+08	1,57	4,23	3,29E-02
CALD1	caldesmon 1	800	1,57	4,40	2,70E-02
ETV1	ets variant 1	2115	1,59	3,92	4,42E-02
MEIS3P1	Meis homeobox 3 pseudogene 1	4213	1,59	5,66	4,35E-02
ANTXR1	anthrax toxin receptor 1	84168	1,59	3,37	4,78E-02
CORIN	corin, serine peptidase	10699	1,60	3,10	3,07E-02
SV2A	synaptic vesicle glycoprotein 2A	9900	1,60	3,54	3,47E-02
RPRD1A	regulation of nuclear pre-mRNA domain containing 1A	55197	1,62	4,28	4,37E-02
TRIL	TLR4 interactor with leucine-rich repeats	9865	1,63	3,78	4,63E-02
TRO	trophinin	7216	1,64	2,85	2,76E-02
DKK3	dickkopf WNT signaling pathway inhibitor 3	27122	1,64	3,16	4,54E-02
FZD3	frizzled class receptor 3	7976	1,65	3,65	4,23E-02
TRIL	TLR4 interactor with leucine-rich repeats	9865	1,68	3,34	3,87E-02
ARHGEF10	Rho guanine nucleotide exchange factor (GEF) 10	9639	1,70	3,55	3,07E-02
ACAA2	acetyl-CoA acyltransferase 2	10449	1,71	7,39	4,78E-02
BHLHE41	basic helix-loop-helix family, member e41	79365	1,80	7,10	4,63E-02
CAPS	calcyphosine	828	1,83	3,63	4,79E-02
TRO	trophinin	7216	1,87	3,18	2,86E-02
CLCN4	chloride channel, voltage-sensitive 4	1183	1,90	3,38	1,35E-02
SCG2	secretogranin II	7857	1,92	3,04	1,14E-02
PDE10A	phosphodiesterase 10A	10846	1,92	3,65	1,51E-02
DUXAP10	double homeobox A pseudogene 10	503639	1,96	4,68	4,78E-02

ST8SIA2	ST8 alpha-N-acetyl-neuraminide alpha-2,8-sialyltransferase 2	8128	2,00	2,82	3,47E-02
MARVELD1	MARVEL domain containing 1	83742	2,00	6,34	4,34E-02
CAPS	calcyphosine	828	2,00	5,24	2,00E-02
CALD1	caldesmon 1	800	2,02	3,51	2,86E-02
CALD1	caldesmon 1	800	2,05	3,76	2,11E-02
ACTA2	actin, alpha 2, smooth muscle, aorta	59	2,10	9,81	4,78E-02
COL8A2	collagen, type VIII, alpha 2	1296	2,15	6,63	4,35E-02
TMTC1	transmembrane and tetratricopeptide repeat containing 1	83857	2,16	5,56	3,92E-02
TNFRSF19	tumor necrosis factor receptor superfamily, member 19	55504	2,19	3,39	1,48E-02
TRO	trophinin	7216	2,20	4,12	2,86E-02
FZD3	frizzled class receptor 3	7976	2,21	4,23	4,11E-02
CALD1	caldesmon 1	800	2,31	6,53	4,78E-02
COL8A2	collagen, type VIII, alpha 2	1296	2,31	6,92	4,35E-02
LTBP2	latent transforming growth factor beta binding protein 2	4053	2,31	8,26	3,87E-02
COL6A1	collagen, type VI, alpha 1	1291	2,39	5,12	3,47E-02
LOXL1	lysyl oxidase-like 1	4016	2,47	8,09	3,47E-02
RGS16	regulator of G-protein signaling 16	6004	2,51	4,77	1,63E-02
ENAH	enabled homolog (Drosophila)	55740	2,51	5,12	4,35E-02
COL6A1	collagen, type VI, alpha 1	1291	2,58	6,09	4,35E-02
FGFR1	fibroblast growth factor receptor 1	2260	2,59	5,48	4,62E-02
FGFR1	fibroblast growth factor receptor 1	2260	2,61	6,88	4,78E-02
BMPR1B	bone morphogenetic protein receptor, type IB	658	2,71	3,39	2,49E-02
SNED1	sushi, nidogen and EGF-like domains 1	25992	2,75	4,61	4,63E-02
SGCD	sarcoglycan, delta (35kDa dystrophin-associated glycoprotein)	6444	2,83	3,96	4,02E-02
BMPR1B	bone morphogenetic protein receptor, type IB	658	2,83	3,05	1,13E-02
LAMP5	lysosomal-associated membrane protein family, member 5	24141	2,87	5,34	2,70E-02
GRP	gastrin-releasing peptide	2922	2,88	3,79	1,48E-02
NTM	neurotrimin	50863	2,95	4,62	8,80E-03
ACTG2	actin, gamma 2, smooth muscle, enteric	72	3,11	5,71	2,15E-02
NTM	neurotrimin	50863	3,13	6,95	4,35E-02
LOC339260	uncharacterized LOC339260	339260	3,27	3,63	5,15E-03
PAX7	paired box 7	5081	3,29	3,16	5,15E-03
COL1A2	collagen, type I, alpha 2	1278	3,40	5,93	4,62E-02
ITGBL1	integrin, beta-like 1 (with EGF-like repeat domains)	9358	3,43	4,48	1,51E-02
ITGBL1	integrin, beta-like 1 (with EGF-like repeat domains)	9358	3,47	5,31	4,02E-02
ITGBL1	integrin, beta-like 1 (with EGF-like repeat domains)	9358	3,61	4,42	4,98E-02
FN1	fibronectin 1	2335	3,76	6,05	2,62E-02
TIMP3	TIMP metallopeptidase inhibitor 3	7078	3,91	8,26	1,48E-02

TIMP3	TIMP metallopeptidase inhibitor 3	7078	3,98	6,84	4,96E-03
**THBS4**	thrombospondin 4	7060	4,00	6,86	3,74E-02
C1QTNF3	C1q and tumor necrosis factor related protein 3	114899	4,23	5,12	2,43E-02
TIMP3	TIMP metallopeptidase inhibitor 3	7078	4,30	7,21	1,86E-02
TIMP3	TIMP metallopeptidase inhibitor 3	7078	4,45	6,66	1,35E-02
FIBIN	fin bud initiation factor homolog (zebrafish)	387758	4,62	5,41	2,62E-02
COMP	cartilage oligomeric matrix protein	1311	5,42	5,14	4,35E-02

Supplementary Table 5: Meta-analysis and two-two comparison of our tumor transcriptomic data with independent publicly available data sets. Our transcriptomic data set was compared to data sets from Slebos *et al*. (2), Mirghani *et al*. (3) and Pyeon *et al*. (4), which allowed the recovery of gene modules named with colors. The Z-score, correlation coefficient (Corr.) and p-value of the two-two comparisons are shown. The modules showing the most genes in common with Cluster 2 are shown in italic.

**Modules**	***vs*. Slebos *et al.***			***vs*. Mirgani *et al.***			***vs*. Pyeon *et al.***		
	**Z-score**	**Corr.**	**p-value**	**Z-score**	**Corr.**	**p-value**	**Z-score**	**Corr.**	**p-value**
Blue				5.34	0.14	0.012			
Red	21.53	0.34	9.4 E^{-07}	*13.62*	*0.38*	*5E^{-08}*	11.56	0.25	0.00029
Brown	10	0.45	5.4E^{-20}	18.07	0.41	4.8 E^{-12}	10.30	0.47	6.4 E^{-15}
Cyan				5.56	0.25	0.014	8.32	0.41	9.7 E^{-5}
Pink				7.42	0.25	0.002	*8.19*	*0.2*	*0.0088*
Yellow							8.10	0.23	0.00054
Turquoise				10.20	0.37	1.1 E^{-17}	6.67	0.22	3.5 E^{-6}
Midnight Blue				5.39	0.21	0.043	*6.05*	*0.21*	*0.054*
Salmon							5.51	0.29	0.0056
Green				12.32	0.46	4 E^{-14}			
Purple	*7.45*	*0.23*	*0.0026*						

Supplementary Table 6: Analysis of the correlation of gene expression with metastasis occurrence. The expression of the *KRT6B*, *S100A7*, *S100A9*, *SERPINB1*, *SPRR1A*, *SPRR1B* and *THBS4* genes was measured by RT-qPCR in patient tumor samples (N=77; Table S4). The median expression (Med) in metastatic (Meta) and non-metastatic (Non-meta) lesions is shown. Median expressions were compared using a two-sample Wilcoxon rank-sum test, and differences were considered statistically significant when $p<0.05$ (shown in bold). A ROC-curve analysis of the relationship between expression and the occurrence of metastatic relapse within 3 years was carried out, and optimal cut-off values were determined. The predictive power of these values was assessed by determining their sensibility, specificity and area under the curve (AUC).

Gene name	RT-qPCR Med. Non-Meta.	RT-qPCR Med. Meta.	p-value	Sensibility	Specificity	AUC
KRT6B	1.01	0.27	0.24	0.84	0.56	0.70
S100A7	1.06	0.13	0.06	0.85	0.56	0.70
S100A9	1.04	0.14	**0.02**	0.94	0.56	0.75
SERPINB1	1.05	0.29	**0.01**	0.93	0.75	0.84
SPRR1A	1.04	0.07	**0.04**	0.83	0.75	0.79
SPRR1B	1.00	1.16	0.78	/	/	/
THBS4	0.95	2.65	**0.02**	0.78	0.72	0.75

2 Supplementary Figure legends

Supplementary Figure 1. The ΔNp63-dependent molecular signature is consistently retrieved in independent transcriptomic data sets.

(A) Flowchart of the WGCNA. Gene co-expression networks (depicted as connected dots) are identified in both data sets. A pairwise comparison and hierarchical clustering analysis of these networks allows the identification of highly interconnected clusters of genes called modules. A dynamic tree cut analysis and dendogram is established for each data set (Figures S1B-G). Their pairwise comparison matching clusters allow the identification of the most preserved ones (see also Table S3). (B-G) Meta-analysis comparing our HPV-positive OSCC transcriptomic data set to independent publicly available data sets was carried out. Gene dendograms and inferred module colors are shown for our data set (B; D; F) and data sets previously reported by Slebos et al. (2) (C), Mirghani et al. (3) (E) and Pyeon et al. (4) (G). Asterisks highlight the purple (B), red (D), and blue and pink modules (E).

Supplementary Figure 2. Modulation of ΔNp63 expression in SCC90 and SCC47 cell line models

(A) Analysis of the ΔNp63 gene (RT-qPCR) and protein (western blot) expression in SCC47 and SCC90 HPV-positive cell line models. RT-qPCR data is shown as scatter plots with bars and mean +/- SEM (N=3). ΔNp63 protein signals were quantified with respect to the actin loading control, and quantification results (p63/Actin) are shown below the blot. (B) Analysis of the migration (left panel) and invasion (right panel) abilities of the SCC90 (upper panels) and SCC47 (lower panels) cells, as evaluated using a trans-well chamber assay. Magnification (100X; 400X) is shown. (C) siRNA-mediated downregulation of ΔNp63 expression in SCC90 cells. (D) Morphology of SCC90 cells upon downregulation of ΔNp63. Magnification (100X) is shown. (E) Analysis of the migration (left panels) and invasion (right panels) properties of SCC90 cells upon ΔNp63 downregulation using a transwell assay. Magnification (100X; 40X) is shown. (F) Western blot analysis of the expression of ΔNp63 in SCC47 cells transfected with a ΔNp63 expression vector. (G) Morphology of ΔNp63-overexpressing SCC47 cells. Magnification (100X) is shown. (H) Analysis of the migration (left panels) and invasion (right panels) properties of SCC47 cells upon ΔNp63 upregulation using a transwell assay. Magnification (100X; 40X) is shown.

Supplementary Figure 3. ΔNp63 is involved in the cellular response to cisplatin and in genotoxic-induced cell apoptosis.

(A-B) Analysis of SCC90 cell survival upon treatment with growing concentrations of cisplatin, using a MTT-based cell viability assay, after transfection with scrambled (siCtrl; A) or anti-ΔNp63 (siΔNp63; B) siRNAs. Results are plotted as sigmoid curves and the cisplatin IC_{50} in both conditions is shown. (C) Morphology of spheroids of SCC90 cells transfected with scrambled or ΔNp63 siRNA and treated with increasing concentration of cisplatin

Supplementary Figure 4. p53 partially mediates cisplatin-induced apoptosis in SCC90 cells.

Western blot analysis of p53, ΔNp63 and cleaved caspase 3 (Cas3*) expression upon siRNA-mediated downregulation of p53 in SCC90 cells and treatment with cisplatin (IC_{50}=2.8µM; IC_{75}=6.7µM). p53 and Cas3* signals were quantified with respect to the actin loading control and normalized to non-treated siCtrl SCC90 cells or siCtrl SCC90 cells treated with the IC_{75} of cisplatin, respectively. Quantification results (p63/Actin; Cas3*/Actin) are shown below the blot.

Supplementary Figure 5. DKK3 does not impact Wnt-signalling in THP-1 macrophages.

(A) Immunocytofluorescence staining of ß-catenin (ß-cat) expression in THP-1 macrophages incubated with DMEM (negative control) or 0.5 µg of hrDKK4 for 6h prior to staining. DAPI, ß-cat staining and merge are shown. Magnification: X200. A magnification (right panels) of the inset in the merge is shown. White and yellow arrowheads highlight ß-cat staining in the cytoplasm and the nuclei, respectively. (B) A quantification of the proportion of THP-1 macrophages with ß-cat-positive nuclei is plotted in a graph. Data is represented as scatter plots with bars and mean +/- SEM (N=3). NS: Non-significant.

Supplementary Figure 6. DKK3 activates NF-κB signaling in THP-1 macrophages.

(A) Immunocytofluorescence staining of p65 expression in THP-1 macrophages incubated with DMEM (negative control), 0.1µg of TNF-α or 0.5 µg of hrDKK4 for 6 h prior to staining. DAPI, p65 staining and merge are shown. Magnification: X200. A magnification (right panels) of the inset in the merge is shown. White and yellow arrowheads highlight p65 staining in the cytoplasm and the nuclei, respectively. (B) Analysis of the expression of the *CCL4*, *CXCL10*, *IL1B* and *IKBA* genes in THP-1 macrophages incubated with DMEM (negative control), 0.1µg of TNF-α or 0.5 µg of hrDKK4 for 30 min and 2 h. Data is represented as as scatter plots with bars and mean +/- SEM (N≥3). One-way ANOVA and Tuckey post-test: * $p<0.05$; **$p<0.01$; *** $p<0.001$; **** $p<0.0001$.

Supplementary Figure 7. CKAP4 is required for the DKK3-dependent activation of NF-κB signaling in THP-1 macrophages.

Immunocytofluorescence staining of p65 expression in THP-1 macrophages transfected with either scrambled (siCtrl) or anti-CKAP4 (siCKAP4) siRNAs, and incubated with DMEM (negative control) or 0.5 µg of hrDKK4 for 6 h prior to staining. DAPI, p65 staining and merge are shown. Magnification: X200. A magnification (right panels) of the inset in the merge is shown. White and yellow arrowheads highlight p65 staining in the cytoplasm and the nuclei, respectively.

Supplemental Video S1. In vitro time-lapse analysis of the phagocytosis of SCC90 cells by THP-1 macrophages

Green-labelled SCC90 cells were co-cultured with red-labelled THP-1 macrophages, cultures were analyzed using a time-lapse video-microscopy approach. Images were acquired every 10min for 22h and 20min using the 20X objective of a IncuCyte® S3 Live-Cell Analysis Instrument.

3 References

1. Barbieri CE, Tang LJ, Brown KA, Pietenpol JA. Loss of p63 leads to increased cell migration and up-regulation of genes involved in invasion and metastasis. Cancer Res. 2006;66(15):7589-97.
2. Slebos RJ, Yi Y, Ely K, Carter J, Evjen A, Zhang X, et al. Gene expression differences associated with human papillomavirus status in head and neck squamous cell carcinoma. Clin Cancer Res. 2006;12(3 Pt 1):701-9.
3. Mirghani H, Ugolin N, Ory C, Lefevre M, Baulande S, Hofman P, et al. A predictive transcriptomic signature of oropharyngeal cancer according to HPV16 status exclusively. Oral Oncol. 2014;50(11):1025-34.
4. Pyeon D, Newton MA, Lambert PF, den Boon JA, Sengupta S, Marsit CJ, et al. Fundamental differences in cell cycle deregulation in human papillomavirus-positive and human papillomavirus-negative head/neck and cervical cancers. Cancer Res. 2007;67(10):4605-19.
